# Characterization of a natural triple-tandem c-di-GMP riboswitch and application of the riboswitch-based dual-fluorescence reporter

**DOI:** 10.1038/srep20871

**Published:** 2016-02-19

**Authors:** Hang Zhou, Cao Zheng, Jianmei Su, Bo Chen, Yang Fu, Yuqun Xie, Qing Tang, Shan-Ho Chou, Jin He

**Affiliations:** 1 State Key Laboratory of Agricultural Microbiology, College of Life Science and Technology, Huazhong Agricultural University, Wuhan, Hubei 430070, People’s Republic of China; 2 Key Laboratory of Fermentation Engineering (Ministry of Education), College of Bioengineering, Hubei University of Technology, Wuhan, Hubei 430068, People’s Republic of China; 3 Institute of Biochemistry, and NCHU Agricultural Biotechnology Center, National Chung Hsing University, Taichung, Taiwan

## Abstract

c-di-GMP riboswitches are structured RNAs located in the 5′-untranslated regions (5′-UTRs) of mRNAs that regulate expression of downstream genes in response to changing concentrations of the second messenger c-di-GMP. We discovered three complete c-di-GMP riboswitches (Bc3, Bc4 and Bc5 RNA) with similar structures, which are arranged in tandem to constitute a triple-tandem (Bc3-5 RNA) riboswitch in the 5′-UTR of the *csp*ABCDE mRNA in *Bacillus thuringiensis* subsp. *chinensis* CT-43. Our results showed that this natural triple-tandem riboswitch controlled the expression of the reporter gene more stringently and digitally than the double-tandem or single riboswitch. A sandwich-like dual-fluorescence reporter was further constructed by fusing the Bc3-5 RNA gene between the two fluorescence protein genes *amcyan* and *turborfp*. This reporter strain was found to exhibit detectable fluorescence color changes under bright field in response to intracellular c-di-GMP level altered by induced expression of diguanylate cyclase (DGC) PleD. Using this system, two putative membrane-bound DGCs from *B. thuringiensis* and *Xanthomonas oryzae* were verified to be functional by replacing *pleD* with the corresponding DGC genes. This report represented the first native triple-tandem riboswitch that was applied to serve as a riboswitch-based dual-fluorescence reporter for the efficient and convenient verification of putative DGC activity *in vivo*.

Riboswitches are *cis*-regulatory RNA elements residing in the 5′-untranslated region (5′-UTR) of bacterial mRNAs that modulate downstream gene expression upon binding of small specific molecules^1^. A typical riboswitch usually contains two domains, an evolutionarily conserved aptamer domain for binding a target metabolite, and an expression platform for regulating downstream gene expression *via* a ligand-dependent conformational swtich^1^. To date, a variety of riboswitches have been characterized to sense a wide variety of ligands, including anions, metal ions, amino acids, sugars, coenzymes, nucleotides and their derivatives (for example c-di-GMP)^1^,^2^. Riboswitch-mediated gene regulation can occur at the stages of transcription, translation, gene splicing, or mRNA stabilization^3^.

In 2008, Breaker’s group reported the first c-di-GMP riboswitch (termed c-di-GMP-I) in eubacteria^4^. Shortly after, they discovered another c-di-GMP riboswitch called c-di-GMP-II in the *Clostridium difficile*^5^. c-di-GMP-I binds the second messenger c-di-GMP through hydrogen bonding or base stacking of the two guanosines of c-di-GMP with five specific bases in the aptamer, resulting in transcriptional changes of downstream genes correlating with c-di-GMP function^4^,^6^,^7^,^8^, whereas c-di-GMP-II is regulated by c-di-GMP-mediated self-splicing to generate a ribosomal binding site involved in translational control^5^,^7^,^8^.

It is noteworthy that riboswitches occasionally exist in tandem mode^1^,^3^,^9^,^10^. Up to date, four natural-occurring double-tandem riboswitches have been reported: 1) A glycine riboswitch contains two similar glycine aptamers and one expression platform in both *Vibrio cholera* and *Bacillus subtilis*^11^. 2) Two complete thiamine pyrophosphate (TPP) riboswitches with similar structures that are adjacent to each other in *B. anthracis*^12^. 3) A SAM-II/SAM-V tandem arrangement riboswitch that senses S-adenosylmethionine (SAM) to allow for both transcriptional and translational control of downstream gene expression in “*Candidatus* Pelagibacter ubique”^13^. 4) Two independent riboswitches that are sequentially arranged to recognize separate ligands of SAM and coenzyme B_12_ in *B. clausii*^14^. All of these riboswitches contain either two aptamers with one expression platform, or possess two independently complete riboswitches. These tandem architectures were found to exhibit more intricate ligand dose-response regulation and was termed “more digital” to allow greater gene expression change triggered by smaller ligand concentration change^1^,^3^,^9^,^10^. For instance, glycine-activated riboswitch containing two separate glycine aptamers can respond cooperatively to sense lower glycine concentration due to the additional interactions with the second aptamer^15^,^16^,^17^ when the first aptamer binds glycine^11^,^18^.

In *B. thuringiensis* subsp. *chinensis* CT-43^19^,^20^, five c-di-GMP riboswitches are annotated in the Rfam database, with three of them arranged in tandem and possibly forming a triple-tandem riboswitch. In addition, each riboswitch is complete with both aptamer and expression platform domains. The complex architecture and potentially sophisticated regulation mechanism of this triple-tandem riboswitch thus deserves more attention.

c-di-GMP is a second messenger that has been implicated in many important cellular processes, such as biofilm formation and dispersion, flagellar motility, adhesion to biotic and abiotic surfaces, virulence, cell-cell signaling, and other physiological processes^21^,^22^,^23^. These phenotypes are induced through binding of c-di-GMP to a variety of specific protein receptors^24^ and c-di-GMP riboswitches^23^. Intracellular c-di-GMP concentration is thus a key issue, which has been regulated by two functionally opposing enzymes, the diguanylate cyclases (DGCs) containing a GGDEF domain, and phosphodiesterases (PDEs) containing either an EAL or HD-GYP domain^25^. Usually many different DGCs and PDEs are present in a bacterium, and not everyone is active simultaneously unless induced by specific temporary or spatial stimulating signals. Yet, current methods of DGC activity characterization are mainly determined by the *in vitro* methods. These methods usually require tedious purification of putative DGC targets and incubation with substrate GTP under a favorable reaction condition, followed by identification of the reaction products by HPLC, LC-MS/MS^26^,^27^ or radiometric TLC^27^,^28^. However, to obtain soluble proteins and to optimize reaction conditions for a successful DGC enzyme assay still impose a great challenge for scientists to date. Alternatively, DGC activity can be monitored *in vivo* through homologous or heterologous expression of certain putative DGC gene to observe the resulting phenotypic difference. Under such a condition, the activity of any putative DGC can be more reliably determined because the reaction is carried out under a more physiological condition. However, some DGCs exhibit low activity and can only produce a minute amount of c-di-GMP that is beyond the detection limit of the current methodology. In addition, most of these phenotypic observation methods, including the Congo red staining^29^,^30^,^31^, motility^31^,^32^,^33^,^34^, aggregation (or sedimentation)^29^,^32^ and biofilm formation^35^,^36^, are indirect, and are subject to the influence of multiple effectors, rendering these methods difficult to accurately measure target DGC activities. Therefore, a direct, sensitive, and more reliable method for DGC activity verification is urgently demanded. A cyclic di-GMP-responsive promoter37 has been used to construct a fluorescent protein reporter for c-di-GMP *in vivo* monitoring. Usually, c-di-GMP-I aptamer exhibits high specificity and strong affinity toward c-di-GMP^4^. The chimera-type^38^ or artificial ribozyme^39^,^40^,^41^ incorporating c-di-GMP-I aptamer as the sensing domain have been applied as the *in vivo* c-di-GMP reporters. However, these reporters are usually instrument-dependent, therefore are inconvenient for routine DGC activity detections.

In this study, we have aimed to solve the above problems by incorporating a triple-tandem Bc3-5 RNA as a new reporter system to more reliably verify putative DGC activities. Herein, we first propose a transcriptional activation model for each single c-di-GMP-I in the triple-tandem arrangements. After characterization of each single unit as well as their various double-tandem arrangements, we confirmed that the triple-tandem architectures exhibited an even larger dynamic range in regulating gene expression. Based on this, we have constructed a novel reporter system comprising the naturally-occurring Bc3-5 fused between two fluorescence genes. This novel reporter system was found to sensitively detect intracellular c-di-GMP level *in vivo* and reliably verify two membrane-bound putative DGCs under regular laboratory conditions without the need for extra expensive instruments.

## Results

### Bioinformatic analyses of the triple-tandem riboswitch and prediction of its regulation mechanism

**Figure 1:**
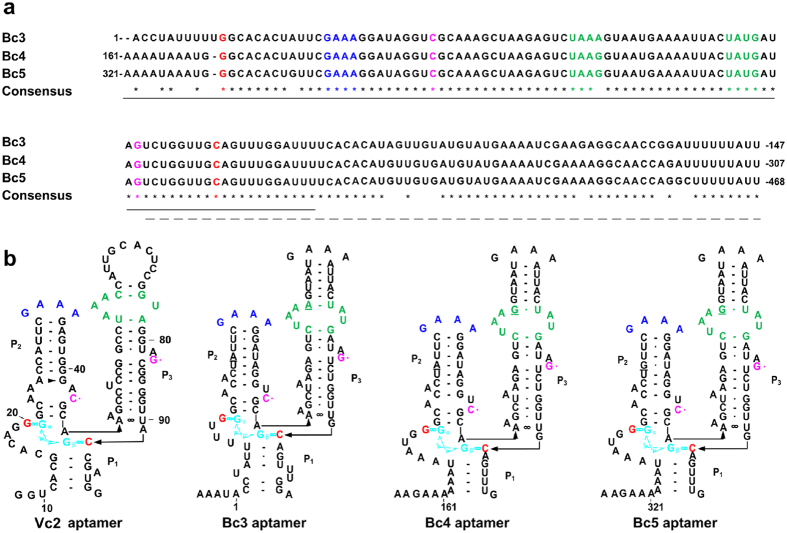
Comparision of primary and secondary structures of Bc3, Bc4 and Bc5 RNA. (**a**) Multiple sequence alignments of Bc3, Bc4 and Bc5 compiled by BioEdit. Aptamers and expression platforms were underlined with solid and dash lines, respectively. (**b**) Secondary structure comparisons of Bc3, Bc4 and Bc5 aptamers with Vc2 aptamer. Conserved motifs such as tetraloop (blue motif in stem P2), tetraloop receptor (green motif in stem P3) and G·C base pair (C base in stem P2 and G base in stem P3 were drawn in magenta) connecting P2 with P3 were all colored to facilitate comparison. c-di-GMP was drawn in cyan and its interacting bases drawn in red.

In the CT-43 genome^19^,^20^ (GenBank accession: NC_017208.1), five type I c-di-GMP riboswitches named Bc1, Bc2, Bc3, Bc4 and Bc5 RNA (Supplementary Tables S1–S5) were annotated in the Rfam database (http://rfam.sanger.ac.uk/search)^42^. While Bc1 and Bc2 are widely distributed in the *B. cereus* group^4^, Bc3, Bc4, and Bc5 are connected in triple-tandem (Bc3-5 see Fig. 1 and supplementary Fig. S1) and only located in the 5′-UTR of the *csp*ABCDE mRNA in some *B. cereus* and *B. thuringiensis* (Supplementary Fig. S2).

Bc3, Bc4 and Bc5 exhibit very high sequence similarities (Fig. 1). Secondary structure predictions by Mfold (http://mfold.rna.albany.edu/?q=mfold)^43^ shows that Bc3, Bc4 and Bc5 share conservative stem-loops and c-di-GMP binding sites similar to Vc2 RNA (Fig. 1b)^44^. Structure prediction and free enthalpy change (Δ*G*) calculation^45^ for Bc4 indicated that the transcription terminator conformation (Supplementary Fig. S3a) was more thermodynamically stable in the absence than in the presence of c-di-GMP (Supplementary Fig. S3b). This suggests that the riboswitch may form a terminator structure that turns off the transcription of downstream genes in the absence of c-di-GMP. In the presence of c-di-GMP, the terminator is not formed because nucleotides in the c-di-GMP binding site (C245), P1 stem (from G247 to U250), and part of P3 stem (from C238 to U243) (Supplementary Fig. S3) become unavailable for interaction. Bc3 and Bc5 share the same mechanism with Bc4. Hence, we assume that Bc3, Bc4 and Bc5 act as three independent c-di-GMP-I elements, and potentially switch on gene transcription in response to c-di-GMP in a cooperative way. Here, we use “read-through rate” of the downstream gene to measure the terminator forming efficiency of a riboswitch.

### Characterization of tandem c-di-GMP-I in *E. coli* strain using *lac*Z as a reporter

In order to test the above assumption, *lac*Z was fused downstream of three truncated 5′-UTR DNA regions containing one, two or three riboswitch elements (*Bc5*′_*lac*Z, *Bc4-5*′_*lac*Z and *Bc3-5*′_*lac*Z) into the plasmid pRP1028 to construct the *lac*Z reporter plasmids. The reporter plasmids were co-transformed into *E. coli* Trans5α with either pET-P*tac*-*pleD* or pET-P*tac* to obtain eight reporter strains (Table 1). These strains were investigated on plates and in liquid medium. The read-through rate of each riboswitch in liquid medium was assessed by β-galactosidase relative specific activity (RSA), which is the ratio of specific activity of a test strain to specific activity of the control strain (lacZ/pET or lacZ/pleD) with the native *lac*Z promoter.

**Figure 2:**
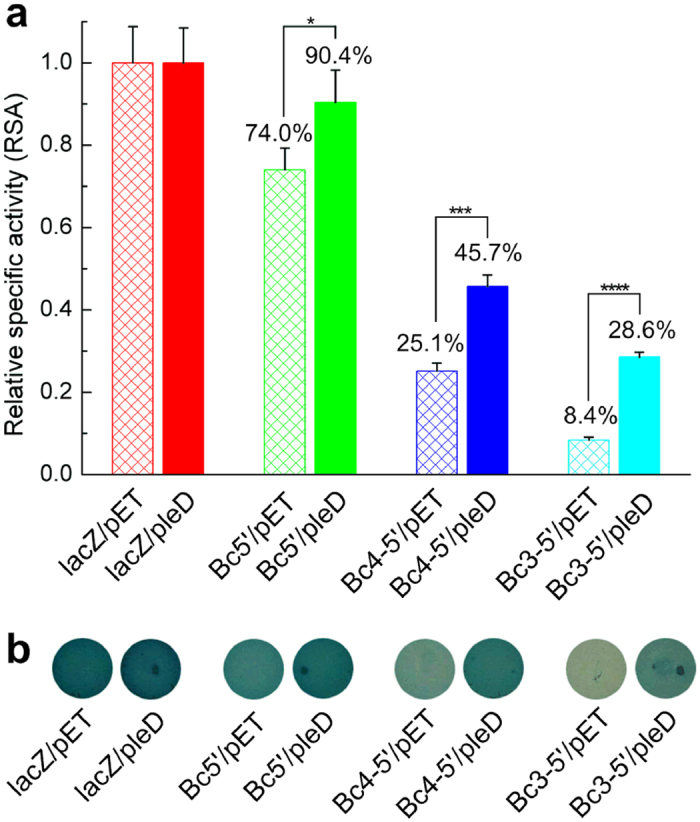
Characterization of tandem c-di-GMP-I in *E. coli* Trans5α using *lacZ* as a reporter. (**a**) Effect of different riboswitches on RSA values. Strains were cultured at 28 °C and β-galactosidase assays were carried out according to the procedure described in the Methods section. RSAs were calculated using formula listed in the Methods section. Data were subjected to one-way analysis of variance (ANOVA) using Bonferroni test by the OriginLab 8.0 software. *P value < 0.05; ***P value < 0.001; ****P value < 0.0001. All Data are averages of three independent experiments (error bars are standard deviation from mean values). (**b**) Blue**-**white spots using the same-batch strains for β-galactosidase assay. These colonies were grown at 28 °C following the procedure in the Methods section. Strain information were listed in Table 1.

Strains lacZ/pleD, Bc5′/pleD, Bc4-5′/pleD, and Bc3-5′/pleD harboring a plasmid pET-P*tac-pleD* constitutively expressed the DGC PleD^46^ and were considered to be in a higher intracellular c-di-GMP concentration state. Conversely, strains lacZ/pET, Bc5′/pET, Bc4-5′/pET, and Bc3-5′/pET carrying an empty vector pET-P*tac* were deemed to contain lower background c-di-GMP levels. As displayed in Fig. 2a, the RSA values of strains containing single, double- and triple-tandem riboswitch were 74.0% (Bc5′/pET), 25.1% (Bc4-5′/pET) and 8.4% (Bc3-5′/pET) respectively under a low c-di-GMP level while the corresponding RSA values were 90.4% (Bc5′/pleD), 45.7% (Bc4-5′/pleD) and 28.6% (Bc3-5′/pleD) respectively under a high c-di-GMP level. These values correspond to increases of 1.2, 1.8 and 3.4 fold from low to high c-di-GMP concentrations. The blue-white spots on X-gal plates correlated well with the RSA values (Fig. 2b). These results suggest that the triple-tandem riboswitch exhibited more stringent gene control (less gene transcription in the absence of c-di-GMP binding) than the double-tandem, and the double-tandem riboswitch was more stringent than the single, which is in accordance with the proposed regulation mechanism.

### Characterization of single, double- and triple-tandem riboswitches in dual-fluorescence reporters

**Figure 3:**
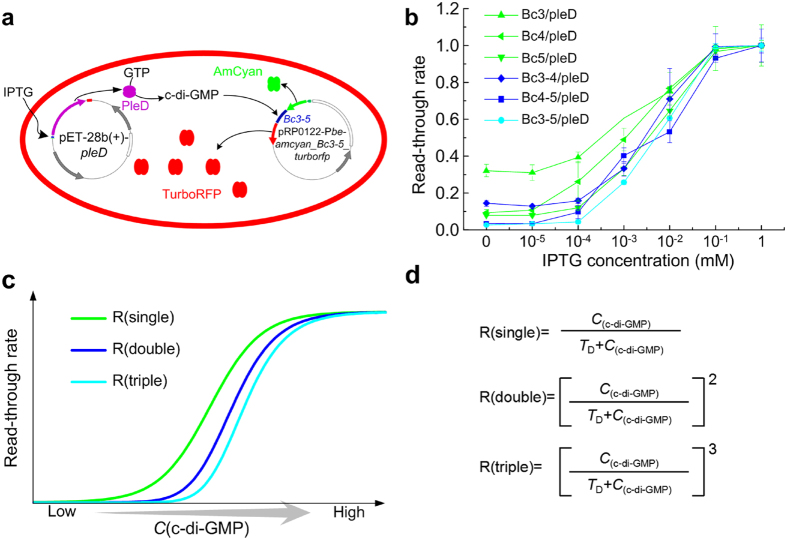
Characterization of tandem c-di-GMP-I using dual-fluorescence reporter in *E. coli* BL21(DE3). (**a**) Schematic diagrams of *amcyan*-*Bc3-5*-*turborfp* sandwich-like dual-fluorescence reporter. IPTG induced expression of PleD (orchid ellipsoid dimer). Accumulating c-di-GMP trigger the c-di-GMP responsive riboswitch to turn on TurboRFP (red ellipsoid dimer) expression, giving rise to TurboRFP fluorescence *versus* the constitutive AmCyan fluoresence (bright green ellipsoid tetramer). (**b**) Read-through rate comparisons among single, double- and triple-tandem c-di-GMP-I. Strains were listed in Table 1. All data were collected from three independent experiments (error bars were standard deviation from mean values). (**c**) The theoretical read-through rate curves of single, double- and triple-tandem c-di-GMP-I. (**d**) The theoretical read-through rate equations of single, double- and triple-tandem c-di-GMP-I. *T*_D_, the equilibrium constant equal to the c-di-GMP concentration (C_(c-di-GMP)_) at 50% read-through rate.

To explore the does-response of these riboswitches on transcription regulation under a tunable intracellular c-di-GMP level, we constructed a new reporter system (Fig. 3a) comprising a series of sandwich-type genetic element cassettes by fusing single, double- and triple-tandem riboswitch DNA in-between the *amcyan* and *turborfp* genes. In this reporter system, addition of IPTG triggers the expression of PleD (Fig. 3a), leading to accumulation of intracellular c-di-GMP. A higher c-di-GMP concentration then activates the sandwiched riboswitch to turn on downstream *turborfp* transcription, resulting with red fluorescence emitted by TurboRFP. As a negative control, strain without addition of IPTG (PleD overexpression is prohibited) will generate little or no c-di-GMP, leading to the conformational change of the riboswitch to the terminator state. Under such a circumstance, only AmCyan would be expressed constitutively.

The relative fluorescence intensity (RFI) is defined as the ratio between the fluorescence intensities of TurboRFP and AmCyan, and this value increases in response to increasing IPTG concentrations (Supplementary Fig. S4). To verify the binding specificity of Bc3, Bc4 and Bc5 with c-di-GMP, the guanine bases (G11 in Bc3, G171 in Bc4 and G331 in Bc5) in the predicted c-di-GMP binding sites were substituted by an adenine base (Fig. 1b). As presented in Supplementary Fig. S4a–c, RFI values of strains Bc3M/pleD, Bc4M/pleD and Bc5M/pleD decrease dramatically under various IPTG concentrations compared with the corresponding wild-type single riboswitch, likely due to the poorer affinity of mutant riboswitch with c-di-GMP than wild-type. The results indicate that Bc3, Bc4 and Bc5 are all transcriptionally active riboswitches.

To characterize transcription regulation of triple-tandem riboswitch (Bc3-5) and double-tandem riboswitch (Bc3-4 and Bc4-5) compared with single riboswitch (Bc3, Bc4 and Bc5), RFI values were measured for the corresponding strains after 20 h induction under different IPTG concentrations. The fold changes of RFI values at different IPTG concentrations ranging from 0 to 1 mM were indicated in Supplementary Fig. S4. Obviously, the triple-tandem riboswitch Bc3-5 (strain Bc3-5/pleD) generated the lowest background RFI (0.036) and highest RFI fold change (about 38 folds). In addition, double-tandem riboswitches also produce better RFI values than single riboswitches. For example, Bc4-5 (Bc4-5/pleD) produces lower background RFI (0.070) and higher RFI fold change (28 folds) than Bc4 (Bc4/pleD, 0.342, 11 folds) or Bc5 (Bc5/pleD, 0.168, 13 folds). Bc3-4 (Bc3-4/pleD, 0.356, 7 folds) presents a similar trend when compared with Bc3 (Bc3/pleD, 0.98, 3 folds). These results were highly correlated with the β-galactosidase assay data shown in Fig. 2.

Supplementary Fig. S4 also shows that RFI values reach a plateau for all riboswitches under 1 mM IPTG concentration, representing a stage of complete read-through expression of *turborfp*. Thus, the RFI value obtained under 1 mM IPTG induction was taken as 100% for each riboswitch. The read-through rate was acquired by calculating the ratio of each RFI value versus the control value of 100% (Fig. 3b). It is easy to see that there is a solid correlation with the theoretical curves (Fig. 3c), which can be generated from equations (Fig. 3d) as supplied by Welz and Breaker^12^. Obviously, tandem architectures yielded more digital dose-response curves, with the triple-tandem riboswitch regulating gene more stringent than the double-tandem riboswitch, which is more stringently than the single riboswitch.

### Reliability assessment of Bc3-5 based dual-fluorescence reporter for sensing c-di-GMP

**Figure 4:**
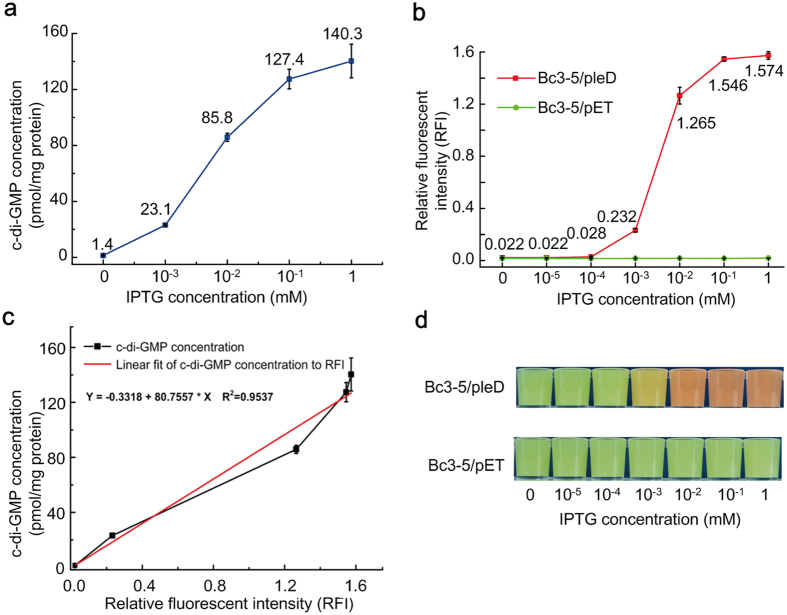
Correlation between intracellular c-di-GMP concentration and RFI. (**a**) Quantification of c-di-GMP concentration of Bc3-5/pleD strain under IPTG induction at different levels. (**b**) RFI values of Bc3-5/pleD and Bc3-5/pET strains under IPTG induction at different levels. (**c**) Correlation between intracellular c-di-GMP concentration and RFI. (**d**) Visible fluorescence of Bc3-5/pleD and Bc3-5/pET concentrated bacterial suspensions under a bright field. Strains were listed in Table 1. Bacterial culture, concentration, resuspension and photography were carried out as described in the Methods section. RFI was calculated as the ratio of fluorescence intensity at 489 nm to fluorescence intensity at 547 nm. All Data were averages of three independent experiments (error bars were standard deviation from mean values).

Because strain Bc3-5/pleD demonstrated the lowest background RFI value and the largest difference in the RFI curve (Supplementary Fig. S4), Bc3-5 DNA was therefore the most optimal regulatory element to serve as the dual-fluorescence reporter. As depicted in Fig. 4a, the intracellular c-di-GMP level (measured by LC-MS/MS) of Bc3-5/pleD ranged from 1.4 pmol/mg to 140.3 pmol/mg when IPTG concentration increases. Hence, a positive correlation between the IPTG concentrations and c-di-GMP levels was observed. Meanwhile, the RFI values of the same-batch bacterial cultures showed a similar dependence on the IPTG concentration (Fig. 4b). Thus, intracellular c-di-GMP level showed a linear correlation with RFI (Fig. 4c). As the Bc3-5/pET culture contains no heterogeneously expressed DGC, the stable and low RFI value indicated that IPTG itself had no effect on RFI (Fig. 4b,d). Therefore, RFI can serve as a reliable alternate to evaluate the intracellular c-di-GMP level.

**Figure 5:**
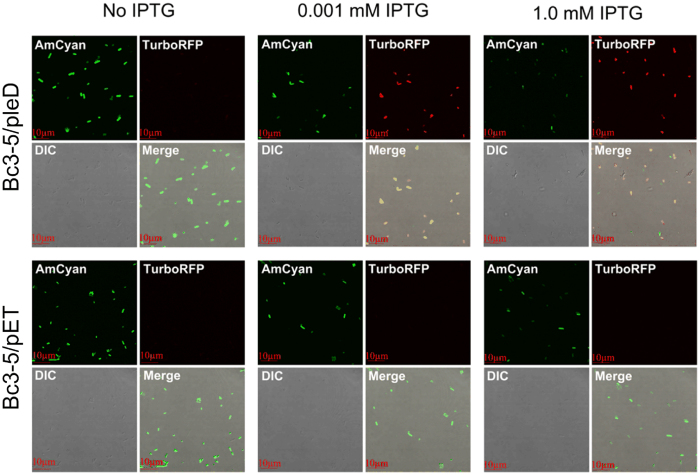
Dual fluorescence imaging of strains Bc3-5/pleD and Bc3-5/pET under IPTG induction. Differential interference contrast (DIC) microscopy images were taken on the same system using a single channel transmission detector. MERGE images represented the overlay of the fluorescence images and the corresponding DIC images.

Although RFI and microscopic fluorescence imaging are considered to be the most straightforward methods for intracellular c-di-GMP monitoring, they might not be always accessible due to their stringent dependency on sophisticated instruments. Thus we have invented an easier-to-use methodology to assess the intracellular c-di-GMP level in bacteria by merely observing the fluorescence intensity under a bright field. As expected, concentrated bacterial suspension of Bc3-5/pleD gradually turned reddish with increasing IPTG concentrations after induction at 28 °C for 20 h, while the control Bc3-5/pET suspension remained green (Fig. 4d). Images captured by confocal laser scanning microscope confirmed this result. Under the 100× oil microscopic field, the TurboRFP fluorescence of strain Bc3-5/pleD was stronger at 1.0 mM IPTG than at 0.01 mM IPTG, while the negative control Bc3-5/pET gave nearly constant green color of the AmCyan fluorescence with little interference from red TurboRFP fluorescence (Fig. 5). It should be further pointed out that the yellow color (Fig. 4d) in the middle Bc3-5/pleD suspension is a mix of the red TurboRFP and green AmCyan proteins within the cells (Fig. 5). So, Bc3-5 based dual-fluorescence system is a feasible and efficient c-di-GMP reporter, which might enable reliable verification of putative DGCs *in vivo*.

### Verification of putative DGCs using the novel Bc3-5 based dual-fluorescence reporter

**Figure 6:**
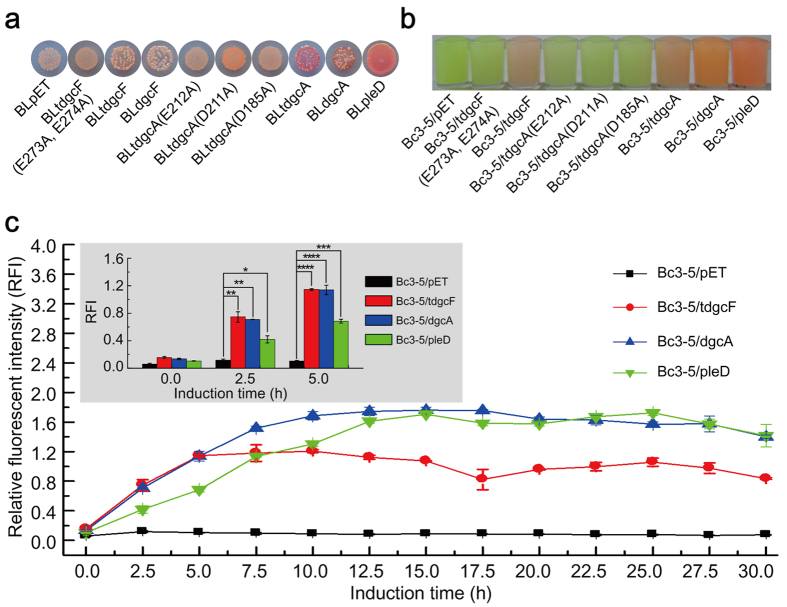
Verification of heterogeneously expressed putative DGCs in *E. coli* BL21(DE3) using Bc3-5 based dual-fluorescence c-di-GMP reporter. (**a**) Verification of putative DGCs by Congo red staining. Strains BLpET and BLpleD were used as negative and positive controls, respectively. (**b**) Verification of putative DGCs by Bc3-5 based dual-fluorescence c-di-GMP reporter strains. Strains Bc3-5/pET and Bc3-5/pleD were used as negative and positive controls, respectively. Bacterial culture, concentration, resuspension and photography of the concentrated bacterial suspensions were carried out as described in the Methods section. (**c**) Verification of putative DGCs *via* RFI measurements. Strains Bc3-5/pET and Bc3-5/pleD were used as negative and positive controls, respectively. Cultures were induced with 1 mM IPTG at 28 °C, and RFI values were measured at different induction time points (0–30 h). Significance analysis was conducted from the RFI values of three induction time points (0 h, 2.5 h and 5 h). Data were subjected to one-way analysis of variance (ANOVA) using Bonferroni test with three measurements by OriginLab 8.0 software. *P value < 0.05; **P value < 0.01; ***P value < 0.001; ****P value < 0.0001. Strains used were listed in Table 1. All Data were averages of three independent experiments (error bars were standard deviation from mean values).

DgcA and DgcF are two putative membrane-bound DGCs harboring a C-terminal GGDEF domain from the Gram-negative strain *Xanthomonas oryzae* pv. *oryzae* KACC 10331 (GenBank accession: NC_006834.1)^47^ and Gram-positive strain *B. thuringiensis* BMB171 (GenBank accession: NC_014171.1)^48^, respectively. Congo red staining is a simple method for DGC activity verification. We thus first tested the enzyme activities in its full, truncated and site-mutated forms by using the popular Congo red staining. The colonies of DgcA and tDgcA expression strains BLdgcA and BLtdgcA did turn reddish similar to the positive control strain BLpleD under a low temperature of 25 °C. The point mutants BLtdgcA(D185A), BLtdgcA(D211A) and BLtdgcA(E212A) failed to change in color (Fig. 6a). The results thus verified DgcA as an active DGC under the *in vivo* condition. However, BLtdgcF (Fig. 6a) and BLdgcF showed no apparent color change under the similar condition (data not shown).

Bc3-5 based dual-fluorescence reporter was then applied to verify these two putative DGCs as well. According to the results in Fig. 6b, strains Bc3-5/dgcA, Bc3-5/tdgcA and Bc3-5/tdgcF expressing DgcA, truncated DgcA and truncated DgcF respectively gave bright orange or red color similar to the positive control strain Bc3-5/pleD, demonstrating that all of them possess genuine DGC activity *in vivo*.

Mutational studies were performed to further confirm the activities of these DGCs (Fig. 6b). When the metal ion binding residue D185 of tDgcA was mutated to alanine, the strain Bc3-5/tdgcA(D185A) lost the capability to synthesize c-di-GMP. Similar loss of the c-di-GMP synthase activity was observed when D211 or E212 residue in the highly conserved GGDEF motif was replaced with alanine (strains Bc3-5/tdgcA(D211A) and Bc3-5/tdgcA(E212A) in Fig. 5). Double variation of the highly conserved GGEEF to GGAAF in tDgcF also resulted in the abolishment of DGC activity in strain Bc3-5/tdgcF(E273A, E274A) (Fig. 5).

The DGC activities of tDgcF and tDgcA were also investigated *in vitro* by HPLC. The chromatogram showed that tDgcF consumed GTP and converted it successfully to c-di-GMP under 10 mM Mg^2+^ concentration *via* the pppGpG intermediate (confirmed by LC/Q-TOF, Supplementary Fig. S5). Similarly, we also identified the c-di-GMP product from the tDgcA reaction system *in vitro* by HPLC (data not shown).

Compared with the commonly used Congo red staining method, this novel triple-tandem riboswitch-based dual-fluorescence c-di-GMP reporter seems to be more reliable for putative DGC verification. As shown in Fig. 6C, the RFI values of all three strains containing overexpressed DGCs increase significantly after 2.5 h IPTG induction, and become stable after 7.5 h compared with that of the negative control Bc3-5/pET. Although the color difference between bacterial cultures is insufficient for photography before 5 h, activity of a putative DGC can still be clearly identified based on its RFI value.

## Discussion

To our knowledge, this is the first report to reveal a natural triple-tandem c-di-GMP riboswitch characteristics. To date, a variety of natural double-tandem riboswitches, such as the glycine riboswitch containing two separated aptamers and a single expression platform^11^, as well as riboswitches for ligands of SAM-II/SAM-V^13^, TPP^12^, AdoCbl^14^, T-box^49^, and M-box^50^,^51^, have been reported. These accumulating tandem riboswitches indicate that, when more stringent or more digital regulation is required, bacteria may take a shortcut by forming a tandem riboswitch to exploit their cooperative interaction to form a tighter regulator instead of generating a completely new riboswitch through long term evolution. The triple-tandem c-di-GMP-I might thus be generated to meet this requirement. This assumption might be supported by the physiological function of the *csp*ABCDE operon (Supplementary Fig. S1). We noticed that *csp*ABCDE operon is merely present within *B. thuringiensis* and *B. cereus* (Supplementary Fig. S2), which are insect and human opportunistic pathogens, respectively. Both pathogens can invade their host from intestines, further adhere to and transmit in their host cells for escaping from the host immune system^52^,^53^. As *csp*ABCDE operon was annotated to encode five cell surface proteins, among which four were predicted to contain a conserved cell-wall anchoring motif (Supplementary Fig. S1), which might take part in adhering to host intestines. Thus, the triple-tandem c-di-GMP-I can guarantee a more rapid and definite switch between adherence and transmission *via* the more stringent and digital control of the transcription of *csp*ABCDE operon. However, further researches are necessary to support this assumption.

By incorporating a tandem structure with a suitable number of certain *cis*-regulatory elements, we may be able to design an even more stringent and digital reporter systems to meet our needs. In fact, Wachsmuth *et al.* have recently improved activation ratio between the induced and uninduced gene activity through the tandem arrangements strategy^54^. Even so, design using more elements is not always a better choice. For example, the maximum RFI value in Supplementary Fig. S4 starts to descend when the number of tandem riboswitch reaches three. Therefore too many tandem repeats might instead decrease RFI value and detection sensitivity. Thus, we have not tried to design an artificial c-di-GMP-I containing more than three tandem repeats, since strains with the natural triple-tandem c-di-GMP-I (Bc3-5) already offers a low RFI background that efficiently turns on red fluorescent emission for sensitive c-di-GMP detection. In addition, the nearly linear correlation between RFI value and c-di-GMP concentration (Fig. 4b) makes it ideal to determine intracellular c-di-GMP in a quantitative scale using the riboswitch-based double-fluorescence report.

Direct verification of putative DGCs under the *in vitro* condition encounters difficulties in at least three aspects: 1) Protein may be difficult to express and purify; 2) The activity of DGCs containing membrane-bound domain (e.g. DgcA in this study) may be difficult to verify when the transmembrane domain was truncated. Such transmembrane domain(s) may possess functions (for example, dimerization) to assist c-di-GMP synthesis. In our system, full-length membrane DGCs can be expressed in bacteria without purification to allow for more reliable verification; and 3) Enzyme reaction conditions *in vitro* may not mimic the true intracellular circumstance of the host strain, causing incorrect conclusion regarding enzyme activity. For example, some specific metal ions (e.g. tDgcF need high concentration Mg^2+^ in this study) or other cofactors may be required for efficient synthesis of c-di-GMP, but are absent in the *in vitro* condition.

Since the discovery of the first DGC^55^, many phenotype-based methods have been developed for DGC verification *in vivo*. Congo red staining^29^,^30^,^31^, motility^31^,^32^,^33^,^34^, aggregation (or sedimentation)^29^,^32^ and biofilm formation^35^,^36^ have been applied for almost a decade and have contributed significantly to the DGC discovery. However, all of these are indirect approaches and unknown effectors may interfere with the experimental results. Our Bc3-5 based reporter worked well in the cases of DgcA, tDgcA and tDgcF verifications, but Congo red staining method seems to be limited in detecting weak DGCs for unknown reasons.

Recently, several detection methods incorporating c-di-GMP riboswitch were also established. Gu *et al.* engineered an allosteric ribozyme by joining the hammerhead ribozyme to a c-di-GMP-I aptamer^39^. After calculating the cleavage fraction of ^32^P-labeled ribozyme, the chimera ribozyme was found to exhibit half-maximal effective and half maximal inhibitory concentrations as low as 90 nM and 180 nM, respectively. They incubated the engineered ribozyme together with the cell lysates to verify three DGCs through self-cleavage fraction calculation. This method works fine but possibly is not applicable for laboratories lacking radioactive isotope handling permit. Later, Nakayama *et al.* reported that one could detect c-di-GMP down to a low level of 320 nM by using a modular aptamer strategy^40^. They used a Vc2 aptamer as the c-di-GMP recognition module to stabilize the sensing module (Spinach RNA), which selectively binds a weakly-fluorescent molecule 3,5-difluoro-4-hydroxybenzylidene imidazolinone (DFHBI) to generate fluorescence. However, this method was carried out *in vitro*, and neededincubation with c-di-GMP for 12 h prior to adding DFHBI. The *in vivo* detection of c-di-GMP by this method has not yet been demonstrated. Kellenberger *et al.* constructed similar Vc2-Spinach biosensors for living cell imaging, in which DGC WspR and its variants were applied for c-di-GMP detection *in vivo*^41^. Lately, an electrochemical biosensor based on c-di-GMP-I riboswitch was developed to monitor c-di-GMP level with a detection limit of 50 nM^56^, and ^32^P A33U Vc2 RNA was constructed for c-di-GMP detection by electrophoretic mobility shift assays^57^. In addition, other kinds of c-di-GMP sensors were developed through transcriptional fusing of defined c-di-GMP responding promoters and downstream reporter genes^58^,^59^. Most of these methods can detect c-di-GMP with good sensitivity but suffer from the limitation of tedious sample treatments before detection, such as bacterial cell lysate extraction^39^,^40^,^58^, long time incubation with c-di-GMP^39^,^40^,^41^, and repeated washing of fluorescent dyes^40^,^41^. Herein, we provided a direct method requiring no pretreatment. This methodology is more advantageous when handling batch samples, such as that for entire putative DGCs screening in any bacterial genome.

As far as we know, two reports on the *in vivo* c-di-GMP monitoring^37^ or genome-scale DGC verification^38^ using a riboswitch-based single-fluorescence reporter have been demonstrated. However, transcription of putative DGCs is dependent on the promoter activity^60^. Hence, fluorescence intensity in a single-fluorescence reporter will depend not only on the background c-di-GMP level but also on the promoter activity. In our dual-fluorescence reporter system, RFI was used to eliminate the potential difference caused by different promoter activity and was particularly suitable for checking authentic activity of putative DGCs. In addition, our dual color system allows easier differentiation of red or yellow color from green (Fig. 6b) than that from weak green to strong green (or weak red to strong red) as in a single color system.

Other than the transcription-based riboswitch reporter, a protein-based c-di-GMP reporter had also been confirmed to be equally good, or even better, in real-time monitoring as a sub-cellular monitoring system^61^. However, this system also suffers from some limitations, such as the protein stability and applicable life span. Although our transcription-based riboswitch reporter lacks the ability for real-time monitoring, it is sufficient for gauging intracellular c-di-GMP levels in a quantitative way (Fig. 4a), and allows verification of putative DGCs *via* visualizing under bright field or *via* RFI measurement without expansive instrument setup.

## Methods

### Strains and culture conditions

The bacterial strains used in this work were listed in Table 1. All different *E. coli* strains were grown in the lysogeny broth (LB) medium or LB agar plates at 28 °C or 37 °C with indicated antibiotics. The concentrations used were: 50 μg/mL for kanamycin and 100 μg/mL for spectinomycin.

Plasmids construction procedures and primers used were described in Supplementary Section C (including Supplementary Table S6 and Supplementary Figs S6–S10).

### Bioinformatic analysis of tandem c-di-GMP-I

CT-43 genome (GenBank accession: NC_017208.1)^19^,^20^ was submitted to Rfam database^42^ for non-coding RNA annotation. Each annotated aptamer (Supplementary Tables S1–S5), together with its downstream terminator, was considered as a putative riboswitch. Hypothetical operon *csp*ABCED were submitted to NCBI conserved domains database for annotation. Distribution of tandem riboswitch encoding region and *csp*ABCED operon were searched by BLAST analysis. Multiple alignments (Fig. 1a) and secondary structure prediction (Fig. 1b) were carried out with BioEdit and Mfold^43^, respectively. Vc2 aptamer^4^,^44^ was used for secondary structure comparison with the Bc3, Bc4 and Bc5 aptamers (Fig. 1b). Free enthalpy change was calculated at 28 °C and 0.17 M NaCl by Mfold^43^.

### Determination of β-galactosidase activity

Strains were grown at 28 °C for 11 h in an orbital shaker at 200 rpm in LB medium containing 50 μg/mL kanamycin and 100 μg/mL spectinomycin (LB/Spec/Kan medium). 1 mL of each culture was separately collected and used for the determination of β-galactosidase activity^62^. The β-galactosidase specific activity was normalized by the total protein content. For the observation of blue-white spots, all cultures at an approximate OD_600_ of 0.8 were applied onto LB/Spec/Kan plates containing 150 μg/mL X-gal, and the plates were incubated at 28 °C for 18 h.

RSA was used for the characteristic comparison of Bc3, Bc4-5 and Bc3-5 using *lacZ* as the reporter. The formulae for RSA calculation are as follows:


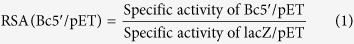



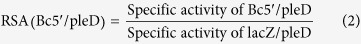



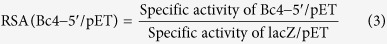



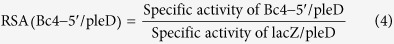



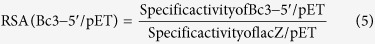



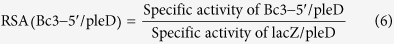


### Determination of RFI

All c-di-GMP reporter strains were grown in LB/Spec/Kan medium at 28 °C and 200 r/min to an OD_600_ of approximately 0.8. After addition of IPTG with a 10-fold increasing gradient ranging from 0.001 mM to 1 mM, the cultures were incubated for 20 h at the same conditions and then stored at 4 °C for subsequent experiments. Fluorescence spectra were recorded with a RF-5301PC fluorescence spectrophotometer (Shimadzu, Kyoto, Japan). Samples were diluted to an OD_600_ of 0.1 with water. RFI was defined as the ratio of fluorescence intensity at 574 nm (TurboRFP) to that at 489 nm (Amcyan).

### Photographing of cultures in bright field

The above cultures were concentrated about 10 fold and resuspended in water. They were later transferred into 96-well plates for recording color intensity with a digital camera.

### Fluorescence imaging of live cells

The above bacterial cultures of Bc3-5/pleD and Bc3-5/pET were stored at 4 °C over 2 h to prevent denaturation of the fluorescence proteins. After centrifugation and resuspension in water, the induced cells were smeared on glass slides and then imaged with FV1000 confocal laser scanning microscope (Olympus, Tokyo, Japan).

### Quantification of c-di-GMP by LC-MS/MS

Detection of c-di-GMP was performed by a Finnigan Surveyor Plus liquid chromatography system followed by a Thermo Scientic TSQ Quantum Ultra EMR tandem mass spectrum system (San Jose, CA, USA). The extraction of intracellular c-di-GMP and the HPLC separation were carried out as previously reported^63^,^64^. c-di-GMP was detected using a selected reaction monitoring mode with the following *m*/*z* transitions: 691.135/135.100 at 51 eV, 691.135/152.200 at 39 eV and 691.135/248.300 at 25 eV. Daughter ion 152.200 was selected as the quantitative ion.

### Congo red staining assay

Strains for overexpression of putative DGCs and their variants (Table 1) were grown at 37 °C and 200 rpm in LB medium with 50 μg/mL kanamycin (LB/Kan) until OD_600_ reach about 0.5. Subsequently, 5 μL cultures were applied onto the LB/Kan plates containing 40 μg/mL Congo red and 0.5 mM IPTG. Colony morphology was examined by visual inspection after 2 day incubation at 25 °C.

### Enzymatic activity assays *in vitro*

Putative DGCs and their variants were expressed and purified from overexpression strains (Table 1) by Ni-NTA agarose (GenScript) according to the manufacturer’s protocol.

A standard reaction mixture containing 100 mM Tris-HCl at pH 7.5, 10 mM MgCl_2_, and 500 μM GTP in a total volume of 100 μL was used to detect the product formation of putative DGCs. The reaction was initiated by adding 10 μM enzyme, and the reaction mixture was incubated at 37 °C for 12 h. The reaction was then terminated by heating the reaction mixture in a boiling water bath for 5 min, followed by centrifuged at 16000 × g for 10 min. The formation of c-di-GMP was determined by separating the reaction mixtures by reverse phase liquid chromatography (LC) either on SHIMADZU Prominence Modular high performance liquid chromatography (HPLC) system (Shimadzu Corporation, Kyoto, Japan) or Liquid chromatography/quadrupole time-of-flight tandem mass spectrometry (LC/Q-TOF). For HPLC, the instrumental parameters and type of C18 column were essentially similar to those described previously^64^,^65^. Nucleotides were separated by isocratic elution mode with a mobile phase of 90% aqueous phase (containing 0.2% ammonium acetate) and 10% methanol. LC/Q-TOF system described below.

### Identification of the intermediate product by LC/Q-TOF

LC/Q-TOF analysis was performed by an Agilent 1260 LC system coupled to an ultrahigh definition quadrupole time-of-flight mass spectrometer Model 6540 equipped with a dual source electrospray ionization ion source (Agilent Technologies, Santa Clara, CA, USA). The sample treatment and LC separation were conducted according to previously described methods^64^,^65^.

Q-TOF parameters used were as follows: ionization mode, positive; capillary voltage: 4000 V; nebulizer pressure: 40 psig; drying gas: 9 L/min; gas temperature: 350 °C; fragmentor voltage: 150 V; skimmer voltage: 65 V; octopole RF Peak voltage: 750 V. accurate LC/MS mass spectra were recorded across the range 100–1000 *m/z* at the MS scan rate 2.0 spectra/s. The data recorded was processed using Agilent MassHunter Qualitative Analysis software. Accurate mass measurements of each peak from the total ion chromatograms were obtained by means of an automated calibrant delivery system using a low flow of a calibrating solution (calibrant solution A, Agilent Technologies), which contains the internal reference masses purine (C_5_H_4_N_4_) at *m/z* 121.0509 and HP-921 [hexakis-(1H, 1H, 3H-tetrafluoro-pentoxy) phosphazene] (C_18_H_18_O_6_N_3_P_3_F_24_) at *m/z* 922.0098.

## Additional Information

**How to cite this article**: Zhou, H. *et al.* Characterization of a natural triple-tandem c-di-GMP riboswitch and application of the riboswitch-based dual-fluorescence reporter. *Sci. Rep.* **6**, 20871; doi: 10.1038/srep20871 (2016).

## Supplementary Material

Supplementary Information

## Figures and Tables

**Table 1:** Bacterial strains used in this study.

Strain name	Characteristics	Source
CT-43	*Bacillus thuringiensis* subsp. *chinensis* CT-43 (GenBank accession: CP001907.1)	19
*Xoo* 10331	*Xanthomonas oryzae* pv. *oryzae* KACC 10331 (GenBank accession: NC_006834.1)	47
BMB171	*Bacillus thuringiensis* BMB171 (GenBank accession: NC_014171.1)	48
Trans5α	*E. coli* Trans5α; F^−^φ80 *lac* ZΔM15 Δ(lacZYA-*arg* F) U169 *end*A1 *rec*A1 *hsd*R17(r_k_^−^,m_k_^+^) *sup*E44λ- *thi* -1 *gyr*A96 *rel*A1 *pho*A.	Beijing TransGen Biotech Co., Ltd
BL21(DE3)	*E. coli* BL21(DE3); protein expression host; F^−^*omp*T *hsd*S_B_(r_B_^−^m_B_^−^) *dcm*(DE3) *gal*λ	Beijing TransGen Biotech Co., Ltd
lacZ/pleD	Trans5α containing pET-P*tac*-*pleD* and pRP1028-*lacZ*	This study
Bc5′/pleD	Trans5α containing pET-P*tac*-*pleD* and pRP1028-*Bc5*′-*lacZ*	This study
Bc4-5′/pleD	Trans5α containing pET-P*tac*-*pleD* and pRP1028-*Bc4-5*′-*lacZ*	This study
Bc3-5′/pleD	Trans5α containing pET-P*tac*-*pleD* and pRP1028-*Bc3-5*′-*lacZ*	This study
lacZ/pET	Trans5α containing pET-28b(+) and pRP1028-*lacZ*	This study
Bc5′/pET	Trans5α containing pET-28b(+) and pRP1028-*Bc5*′-*lacZ*	This study
Bc4-5′/pET	Trans5α containing pET-28b(+) and pRP1028-*Bc4-5*′-*lacZ*	This study
Bc3-5′/pET	Trans5α containing pET-28b(+) and pRP1028-*Bc3-5*′-*lacZ*	This study
Bc3/pleD	BL21(DE3) containing pET-28b(+)-*pleD* and pRP0122-P*be*-*amcyan*_*Bc3*_*turborfp*	This study
Bc3M/pleD	BL21(DE3) containing pET-28b(+)-*pleD* and pRP0122-P*be*-*amcyan*_*Bc3M*_*turborfp*	This study
Bc4/pleD	BL21(DE3) containing pET-28b(+)-*pleD* and pRP0122-P*be*-*amcyan*_*Bc4*_*turborfp*	This study
Bc4M/pleD	BL21(DE3) containing pET-28b(+)-*pleD* and pRP0122-P*be*-*amcyan*_*Bc4M*_*turborfp*	This study
Bc5/pleD	BL21(DE3) containing pET-28b(+)-*pleD* and pRP0122-P*be*-*amcyan*_*Bc5*_*turborfp*	This study
Bc5M/pleD	BL21(DE3) containing pET-28b(+)-*pleD* and pRP0122-P*be*-*amcyan*_*Bc5M*_*turborfp*	This study
Bc3-4/pleD	BL21(DE3) containing pET-28b(+)-*pleD* and pRP0122-P*be*-*amcyan*_*Bc3-4*_*turborfp*	This study
Bc4-5/pleD	BL21(DE3) containing pET-28b(+)-*pleD* and pRP0122-P*be*-*amcyan*_*Bc4-5*_*turborfp*	This study
Bc3-5/pleD	BL21(DE3) containing pET-28b(+)-*pleD* and pRP0122-P*be*-*amcyan*_*Bc3-5*_*turborfp*	This study
Bc3-5/pET	BL21(DE3) containing pET-28b(+) and pRP0122-P*be*-*amcyan*_*Bc3-5*_*turborfp*	This study
Bc3-5/dgcA	BL21(DE3) containing pET-28a(+)-*dgcA* and pRP0122-P*be*-*amcyan*_*Bc3-5*_*turborfp*	This study
Bc3-5/tdgcA	BL21(DE3) containing pET-28b(+)-*tdgcA* and pRP0122-P*be*-*amcyan*_*Bc3-5*_*turborfp*	This study
Bc3-5/tdgcA(D185A)	BL21(DE3) containing pET-28b(+)-*tdgcA(D185A)* and pRP0122-P*be*-*amcyan*_*Bc3-5*_*turborfp*	This study
Bc3-5/tdgcA(D211A)	BL21(DE3) containing pET-28b(+)-*tdgcA(D211A)* and pRP0122-P*be*-*amcyan*_*Bc3-5*_*turborfp*	This study
Bc3-5/tdgcA(E212A)	BL21(DE3) containing pET-28b(+)-*tdgcA(E212A)* and pRP0122-P*be*-*amcyan*_*Bc3-5*_*turborfp*	This study
Bc3-5/tdgcF	BL21(DE3) containing pET-28b(+)-*tdgcF* and pRP0122-P*be*-*amcyan*_*Bc3-5*_*turborfp*	This study
Bc3-5/tdgcF(E273A, E274A)	BL21(DE3) containing pET-28b(+)*-tdgcF(E273A, E274A)* and pRP0122-P*be*-*amcyan*_*Bc3-5*_*turborfp*	This study
BLpleD	BL21(DE3) containing pET-28b(+)-*pleD*	This study
BLpET	BL21(DE3) containing pET-28b(+)	This study
BLdgcA	BL21(DE3) containing pET-28a(+)-*dgcA*	This study
BLtdgcA	BL21(DE3) containing pET-28b(+)-*tdgcA*	This study
BLtdgcA(D185A)	BL21(DE3) containing pET-28b(+)-*tdgcA(D185A)*	This study
BLtdgcA(D211A)	BL21(DE3) containing pET-28b(+)-*tdgcA(D211A)*	This study
BLtdgcA(E212A)	BL21(DE3) containing pET-28b(+)-*tdgcA(E212A)*	This study
BLdgcF	BL21(DE3) containing pET-28b(+)-*dgcF*	This study
BLtdgcF	BL21(DE3) containing pET-28b(+)-*tdgcF*	This study
BLtdgcF(E273A, E274A)	BL21(DE3) containing pET-28b(+)*-tdgcF(E273A, E274A)*	This study
