# Supplementary material for: Characterization of a natural triple-tandem c-di-GMP riboswitch and application of the riboswitch-based dual-fluorescence reporter
Source: Sci Rep. 2016 Feb 19;6:20871. doi: 10.1038/srep20871 (PMC4759541; doi:10.1038/srep20871)
Supplement: Supplementary Information [file srep20871-s1.pdf]

Supplementary Information

Characterization of a natural triple-tandem c-di-GMP riboswitch and application of the riboswitch-based dual-fluorescence reporter

Hang Zhou[1,§], Cao Zheng[1,§], Jianmei Su[1,§], Bo Chen[1], Yang Fu[1], Yuqun Xie[2], Qing Tang[1], Shan-Ho Chou[3], Jin He[1,*]

[1]State Key Laboratory of Agricultural Microbiology, College of Life Science and Technology, Huazhong Agricultural University, Wuhan, Hubei 430070, People's Republic of China.

[2]Key Laboratory of Fermentation Engineering (Ministry of Education), College of Bioengineering, Hubei University of Technology, Wuhan, Hubei 430068, People's Republic of China.

[3]Institute of Biochemistry, and NCHU Agricultural Biotechnology Center, National Chung Hsing University, Taichung, Taiwan

[§]These authors contributed equally.

*Correspondence and requests for materials should be addressed to J. H. (E-mail: hejin@mail.hzau.edu.cn)

Section A: Rfam annotation of c-di-GMP-I in *Bacillus thuringiensis* subsp. *Chinensis* CT-43 genome (GenBank accession: NC_017208.1)

Table S1. Bc1 aptamer annotation

**Id**	**Accession**	**Location**	**Bits score**	**E-value**	**Strand**
**c-di-GMP-I**	**RF01051**	**421335..421468**	**72.1**	**1.3e-14**	**+**

Alignment

```
#NC                            v          v             v                              v
#SS                ::::::::::::<<---<<<<<<____>>>>>>--->>--<<<<-<.<<<<<----<<<<____>>>>--->>>-->>>>>>>::::::
#CM              1 uaaugaaAAaGGCAAAcccgccGAAAggcgggGACGCAAAgCcac.gGguCUAAggcccgaaagggcuAuGacAGCcggGcUGCCgaa 87
#MATCH             +A +GA AAAGGCAAAC: :::GAAA::: :GGACGCAAA:C:AC GG::CUAAGG C GAAA G CUA G::AGCC :G:U+CCG A
#SEQ        421335 CAGCGAGAAAGGCAAACUGAUGGAAACAUGAGGACGCAAAACUACaGGAGCUAAGGUC-GAAA-GGCUACGCUAGCC-AGUUACCGGA 421468
#PP                **********************************************************.9999.*************.**********
```

Table S2. Bc2 aptamer annotation

**Id**	**Accession**	**Location**	**Bits score**	**E-value**	**Strand**
**c-di-GMP-I**	**RF01051**	**1087308..1087400**	**59.2**	**1.1e-11**	**+**

Alignment

```
#NC
#SS                :::::::::::<<---<<<<<<____>>>>>>--->>--<<<<-<<<<<<----<<<<.____.....>>>>--->>>-->>>>>>>::::::
#CM              1 uaaugaaAAaGGCAAAcccgccGAAAggcgggGACGCAAAgCcacgGguCUAAggccc.gaaa.....gggcuAuGacAGCcggGcUGCCgaa 87
#MATCH             + AU ++    GCA AC::::CGAAAG::::GG CGCAAA:C:+:G::UCUA GG::: + A      :::CUA+GA: G:C::G:UGC  A
#SEQ       1087308 AGAUAUUUUUAGCACACUAUUCGAAAGGAUAGGCCGCAAAGCUUAGAGUCUACGGUAAuACAUauuggUUACUAAGAUCGUCUGGUUGCACAU 1087400
#PP                **********************************************************6444479999*************************
```

Table S3. Bc3 aptamer annotation

Id	Accession	Location	Bits score	E-value	Strand
**c-di-GMP-I**	**RF01051**	**c(4753101..4753189)**	**72.6**	**3.8e-14**	**-**

Alignment

```
#NC
#SS                 :::::::::::<<---<<<<<<____>>>>>>--->>--<<<<-<<<<<<----<<<<..____>>>>--->>>-->>>>>>>::::::
#CM               1 uaaugaaAAaGGCAAAcccgccGAAAggcgggGACGCAAAgCcacgGguCUAAggccc..gaaagggcuAuGacAGCcggGcUGCCgaa 87
#MATCH              +  U ++   GGCA AC::::CGAAAG::::GG CGCAAA:C:A:G::UCUAA+G:::  +AAA:::CUAUGA:AG:C::G:UGC G
#SEQ        4753189 ACCUAUUUUUGGCACACUAUUCGAAAGGAUAGGUCGCAAAGCUAAGAGUCUAAAGUAAugAAAAUUACUAUGAUAGUCUGGUUGCAGUU 4753101
#PP                 **********************************************************966666*************************
```

Table S4. Bc4 aptamer annotation

Id	Accession	Location	Bits score	E-value	Strand
**c-di-GMP-I**	**RF01051**	**c(4752941..4753029)**	**77.3**	**2.9e-15**	**-**

Alignment

```
#NC
#SS                 :::::::::::<<---<<<<<<____>>>>>>--->>--<<<<-<<<<<<----<<<<..____>>>>--->>>-->>>>>>>::::::
#CM               1 uaaugaaAAaGGCAAAcccgccGAAAggcgggGACGCAAAgCcacgGguCUAAggccc..gaaagggcuAuGacAGCcggGcUGCCgaa 87
#MATCH              +AA+ AAA  GGCA AC::::CGAAAG::::GG CGCAAA:C:A:G::UCUAAGG:::  +AAA:::CUAUGA:AG:C::G:UGC G
#SEQ        4753029 AAAAUAAAUGGGCACACUAUUCGAAAGGAUAGGUCGCAAAGCUAAGAGUCUAAGGUAAugAAAAUUACUAUGAUAGUCUGGUUGCAGUU 4752941
#PP                 **********************************************************966666*************************
```

Table S5. Bc5 aptamer annotation[a)]

Id	Accession	Location	Bits score	E-value	Strand
**c-di-GMP-I**	**RF01051**		**75.4**	**8.3e-15**	**-**

Alignment

```
#NC
#SS                ::::::::::::<<---<<<<<<____>>>>>>--->>--<<<<-<<<<<<----<<<<..____>>>>--->>>-->>>>>>>::::::
#CM             1  uaaugaaAAaGGCAAAcccgccGAAAggcgggGACGCAAAgCcacgGguCUAAggccc..gaaagggcuAuGacAGCcggGcUGCCgaa 87
#MATCH             +AA+ AAA  GGCA AC: ::CGAAAG:: :GG CGCAAA:C:A:G::UCUAAGG:::  +AAA:::CUAUGA:AG:C::G:UGC G
#SEQ               AAAAUAAAUGGGCACACUGUUCGAAAGGAUAGGUCGCAAAGCUAAGAGUCUAAGGUAAugAAAAUUACUAUGAUAGUCUGGUUGCAGUU
#PP                ***********************************************************966666************************
```

[a)] Bc5 RNA was omitted (see Supplementary Figure S1) in GenBank record (GenBank accession: NC_017208.1).

Section B: Additional result figures

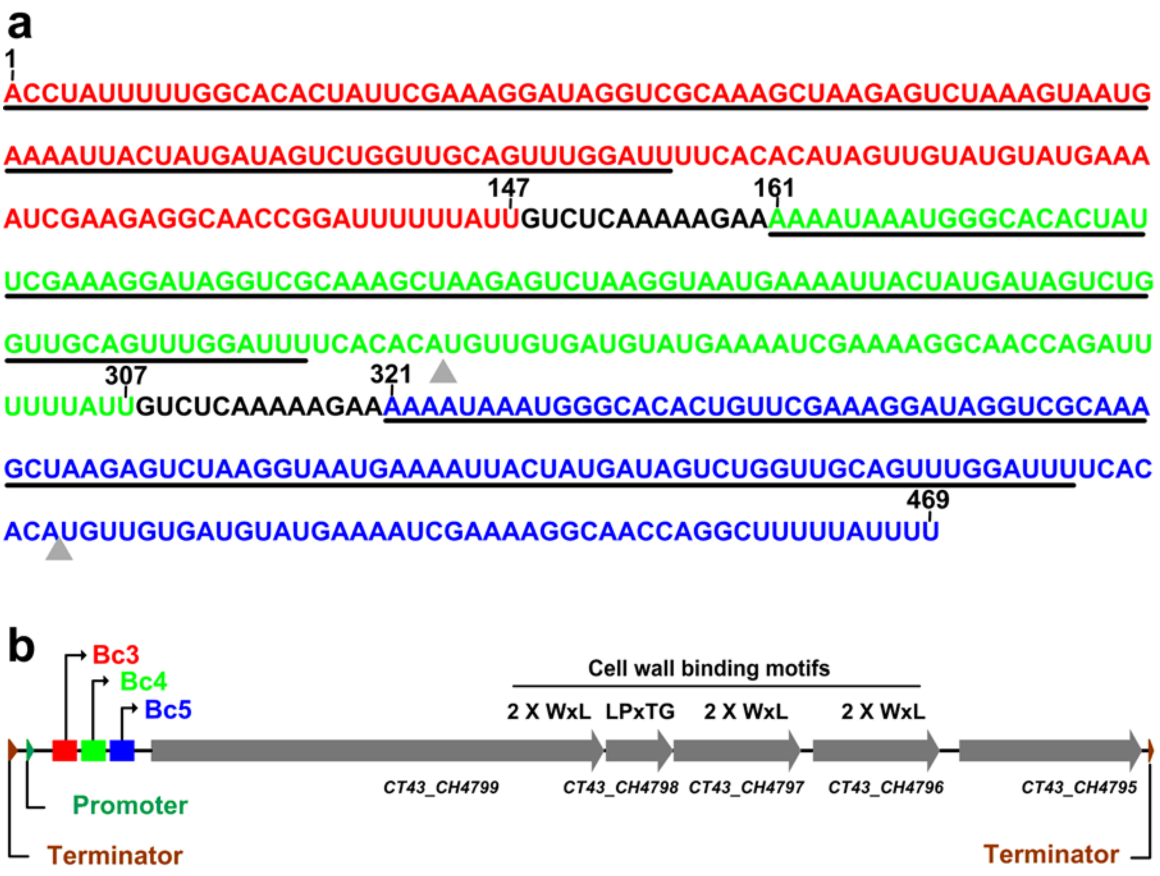

Figure S1. Sequence of triple-tandem c-di-GMP-I encoding region and its downstream operon. (a) Sequence of the triple-tandem c-di-GMP-I Bc3, Bc4 and Bc5 RNA were shown in red, lime and blue colors, respectively. Linkers between the adjacent riboswitches were shown in black color. Aptamer sequences were underlined by solid lines, and the region between two gray triangles was an omission in GenBank record (GenBank accession: NC_017208.1) due to a splicing error. **(b)** Scheme of the triple-tandem c-di-GMP-I encoding region and its downstream operon. Red, lime and blue rectangles were encoding regions of Bc3, Bc4 and Bc5 RNA, respectively. WxL[1] and LPxTG[2] were cell-wall binding motifs. Open reading frames *CT-43_CH4799* (*cspA*), *CT-43_CH4798* (*cspB*), *CT-43_CH4797* (*cspC*), *CT-43_CH4796* (*cspD*), and *CT-43_CH4795* (*cspE*) were annotated as cell surface protein encoding genes in the NCBI protein database (GenBank accession: CP001907.1).

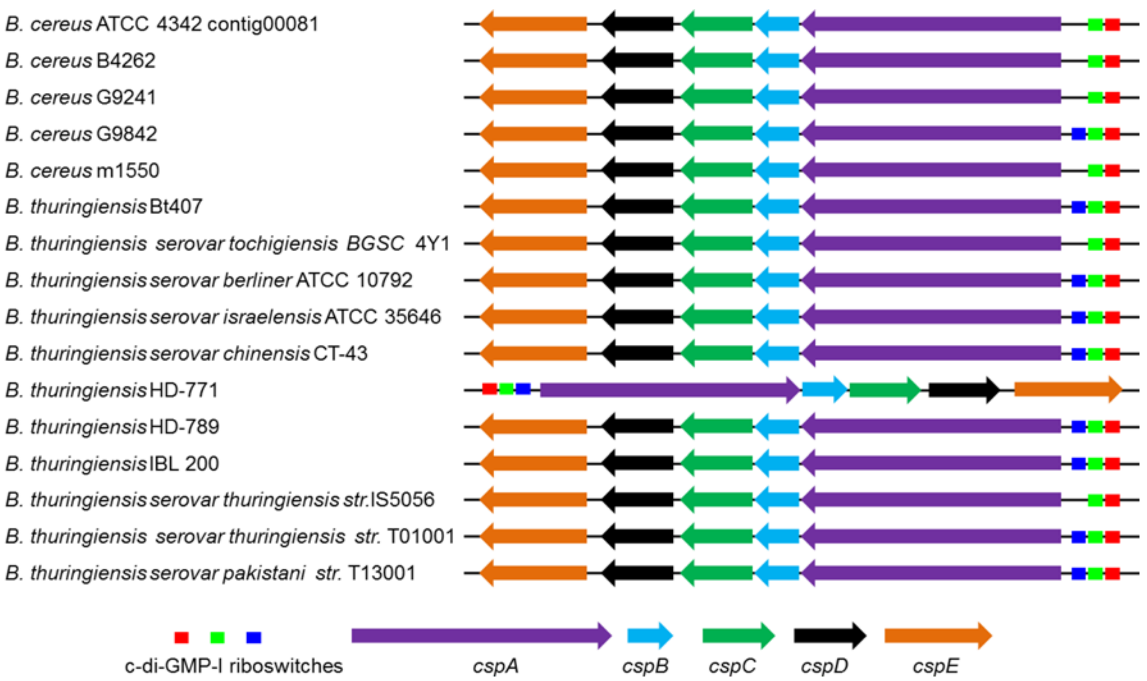

Figure S2. Genetic organization of c-di-GMP-I riboswitch encoding region and its downstream *cspABCDE* operon in *B. cereus* and *B. thuringiensis* strains. Tandem (double- or triple-) c-di-GMP-I riboswitch is usually located in the 5'-UTR of *cspABCDE* transcript (mRNA) in *B. cereus* and *B. thuringiensis* strains**.** It is worth noting that the double-tandem riboswitches may be triple-tandem configuration, which might be caused by a splicing error similar to that in *B. thuringiensis* subsp. *chinensis* CT-43 strain (Figure S1). The conserved genes and riboswitch encoding regions were shown with the different colors, and their names are given in the bottom. Apart from the operon in *B. thuringiensis* HD-771 (positive strand, rightward), all other c-di-GMP-I riboswitches are present in the negative strand (leftward) for the *B. cereus* and *B. thuringiensis* strains.

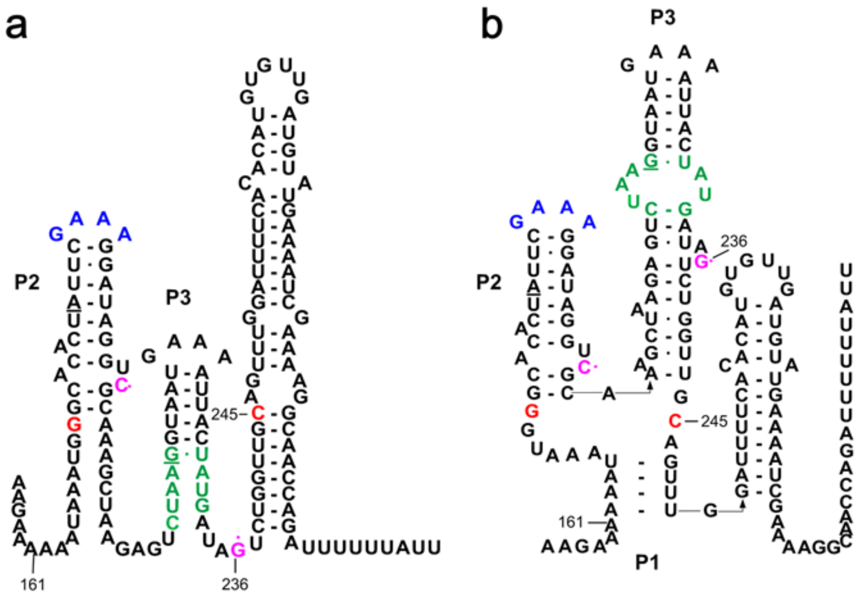

Figure S3. Schematic diagram of a hypothetical allosteric regulation mechanism of Bc4 RNA. (a) The "OFF" conformation (neutral state) containing a transcription terminator is more thermodynamically stable (lower free enthalpy change (*ΔG*) value: -49.10 kcal/mol) than the "ON" conformation. **(b)** The "ON" conformation after c-di-GMP binding leads to a higher *ΔG* value: -30.66 kcal/mol. Conserved motifs, G·C base pair and c-di-GMP binding bases were colored the same as in Figure 1. The *ΔG* values for the corresponding Bc3 RNA in the "ON" and "OFF" conformations are -27.21 and -48.69 kcal/mol, respectively. Similarly, *ΔG* values of the "ON" and "OFF" conformations for Bc5 RNA, are -30.29 and -52.77 kcal/mol, respectively. *ΔG* values were calculated by Mfold at 28°C and 0.17 M NaCl.

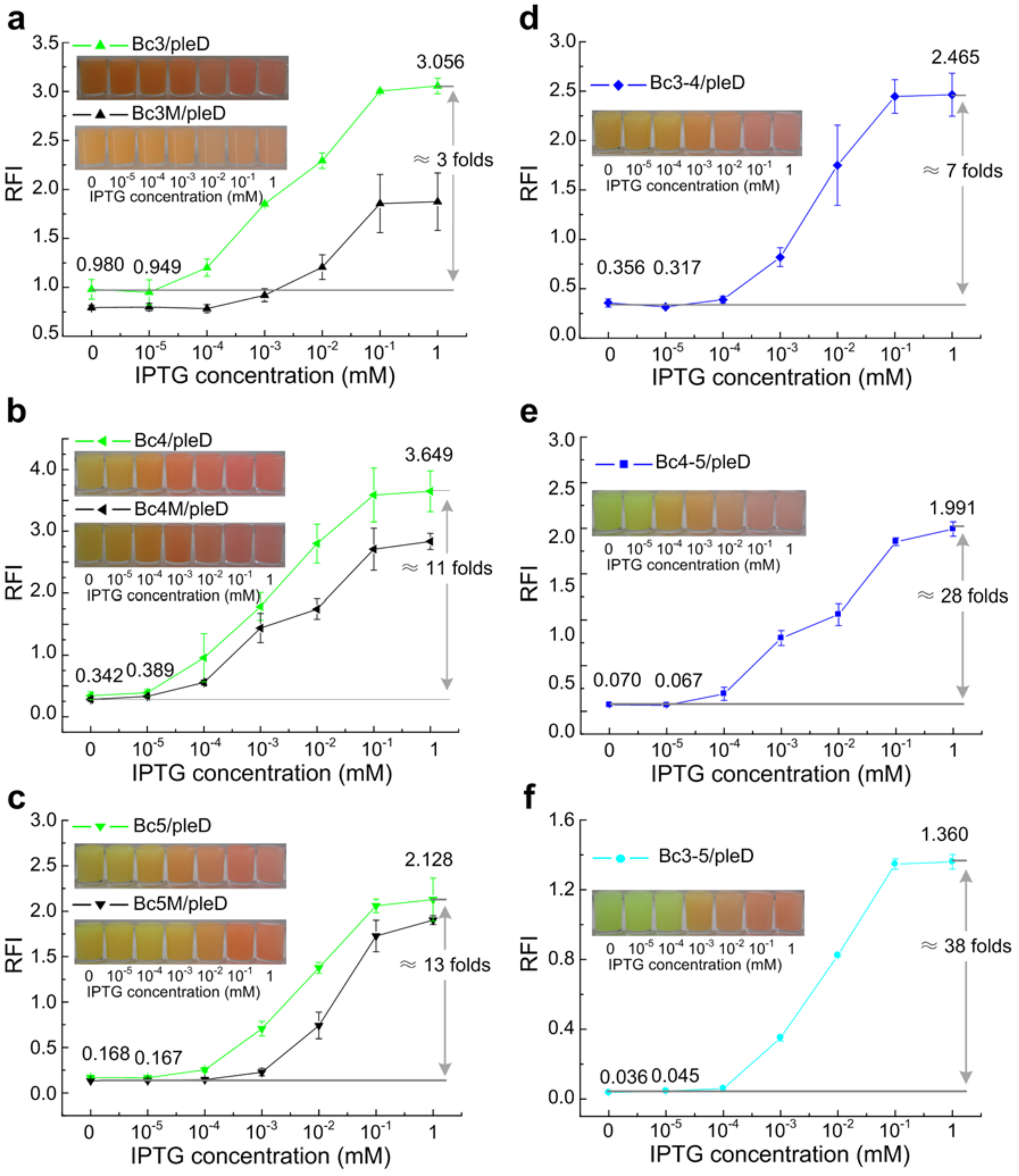

Figure S4. Characterization of different c-di-GMP-I using the dual-fluorescence reporter in *E. coli* BL21(DE3). Cultures (at an OD_{600} of approximately 0.8) were induced with IPTG ranging from 0 to 1 mM at 28°C for 20 h. Photography for concentrated bacterial suspension was carried out as described in the Methods section. The ratio of RFI value in 1 mM IPTG to RFI value in 0 mM IPTG represented the fold change of RFI.

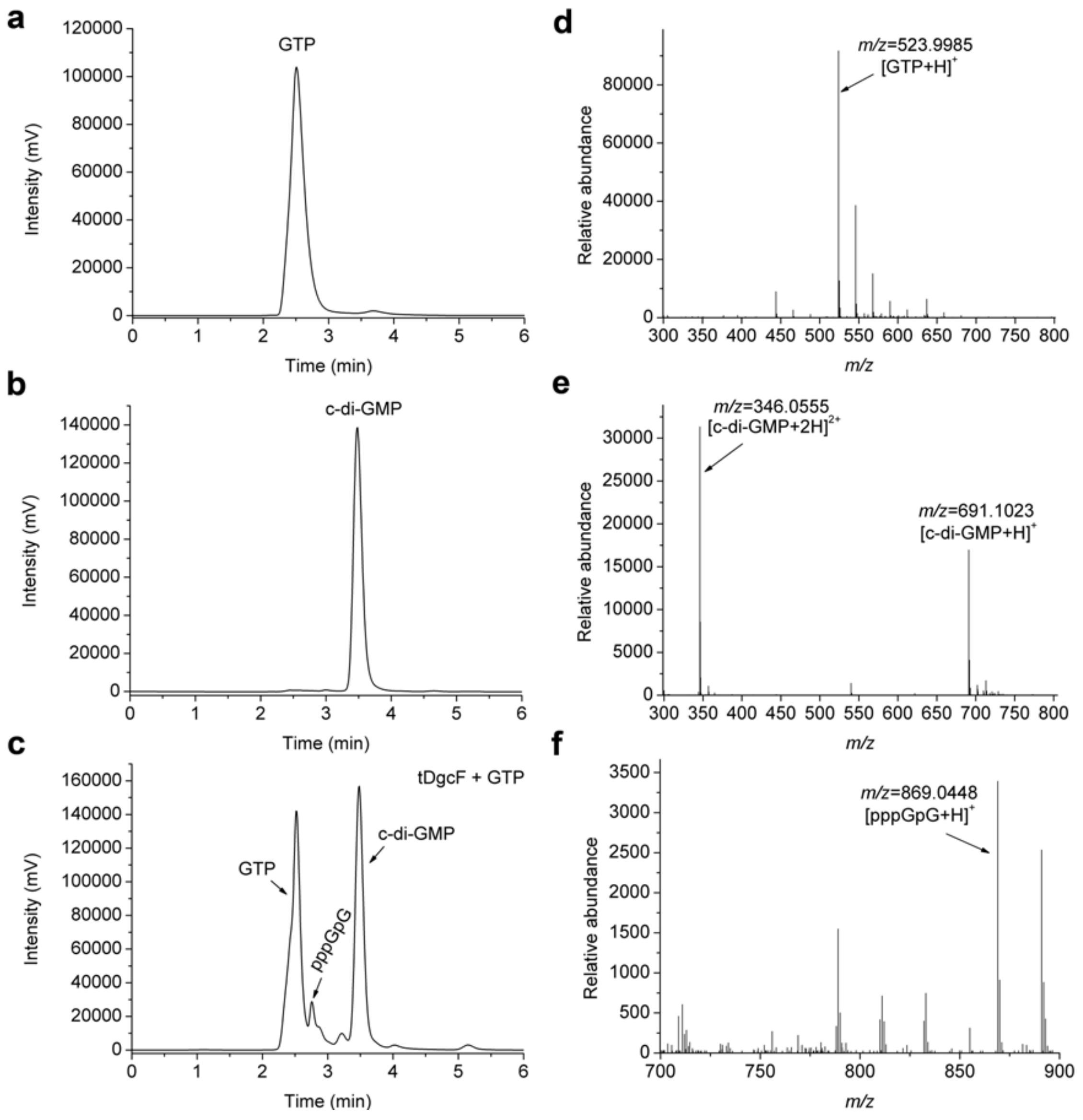

Figure S5. Identification of *in vitro* reaction intermediate of tDgcF by LC/Q-TOF. (a), **(b)** and **(c)** were the HPLC chromatograms of the GTP standard, c-di-GMP standard and *in vitro* reaction products of tDgcF, respectively. **(d)**, **(e)** and **(f)** were the mass spectrums of GTP, c-di-GMP and pppGpG (identified as *in vitro* reaction products), respectively. The *in vitro* reaction, HPLC conditions and LC/Q-TOF were carried out as the described in the Methods section.

Section C: Primers information and plasmids construction procedure.

Table S6. Primers used in this study.

Primer name	Primer sequence [a)]
*Bc5'*_F	CATG***CCATGG***TCTCAAAAAGAAAAAATAAATGGG
*5' UTR*_R	CG***GGATCC***ACTTCTTATGAACCGAATATTTCGAAC
*Bc4'*_F	CATG***CCATGG***TCTCAAAAAGAAAAAATAAATGGG
*Bc3'*_F	CATG***CCATGG***CACGATAAATAAATACCTATTTTTGG
P*tac*_F	GA***AGATCT***GTGCAGGTCGTAAATCACTG
P*tac*_R	GC***TCTAGA***CTCCACACATTATACGAGCCGATG
P*be*_F	CCC***AAGCTT***GGCAAAACAACTTGAAAAAAGTTGTTGAC
P*be*_R	CATGGAAAATTCCTCC***TCTAGA***CTTTCTAAAATTCTTATCATTATAAGTTTC
*amcyan*_F	GAAAG***TCTAGA***GGAGGAATTTTCCATGGCTCTTTCAAACAAGTTTATCGG
*amcyan*_R	GGTATTTATTTATCGT***GGATCC***TTATCAGAAAGGGACAACAGAGGTTATAT GTG
*Bc3*_F	CTGATAA***GGATCC***ACGATAAATAAATACCTATTTTTGGCACAC
*Bc3*_MF	CG***GGATCC***ACGATAAATAAATACCTATTTTTAGCACACTATTCG
*Bc3*_R	CG***GTCGAC***AATAAAAAAATCCGGTTGCCTCTTCG
*Bc4*_F	CG***GGATCC***GTCTCAAAAAGAAAAAATAAATGGGCACACTATTCG
*Bc4*_MF	CG***GGATCC***GTCTCAAAAAGAAAAAATAAATGAGCACACTATTCG
*Bc4*_R	CG***GTCGAC***AATAAAAAAATCTGGTTGCCTTTTCG
*Bc5*_F	CG***GGATCC***GTCTCAAAAAGAAAAAATAAATGGGCACACTGTTCG
*Bc5*_MF	CG***GGATCC***GTCTCAAAAAGAAAAAATAAATGAGCACACTGTTCG
*Bc5*_R	CATGGAAAATTCCTCCTATCTGTTTT***GTCGAC***AAAATAAAAAGCCTGGTTG CCTTTTCG
*turborfp*_F	CAAAACAGATAGGAGGAATTTTCCATGGGAAGTGAATTGATTAAAGAAA ATATGCATATG
*turborfp*_R	GG***GGTACC***ACTAGTAAGCTT***CTCGAG***TTATCTATGCCCTAATTTACTAGG
*pleD*_F	GC***TCTAGA***AATAATTTTGTTTAACTTTAAGAAGGAGATATACCATGAGCGC CCGGATCCTCG
*pleD*_R	CTT***CTCGAG***GGAGCTCGAATTATTAGGCGGCCTTGCCGACCAC
*speR*_F	CTAG***CAGCTG***TTGACAATTAATCATCCGG
*speR*_R	G***GAATTC***CAGA***AGATCT***TCAAGTTGTTTTGCCATCAATTGAGAGAAG
*dgcA*_F	AG***GGATCC***ATGCGCTTGTACGCCGGAC

*dgcA*_R	TGC***<u>AAGCTT</u>***TCAGGTCACCTGATG
*tdgcA*_F	TTGT***<u>CCATGG</u>***GCCTGGCACCGTTGCGC
*tdgcA*_R	TGC***<u>CTCGAG</u>***GGTCACCTGATGACCG
*tdgcA*_185MF	TCTGGCCGGCGCTCGCGTGCT
*tdgcA*_185MR	AGCACGCGAGCGCCGGCCAGAT
*tdgcA*_211MF	CTGGGGCGGCGCAGAATTCCTGATT
*tdgcA*_211MR	AATCAGGAATTCTGCGCCGCCCCAG
*tdgcA*_212MF	TGGGGCGGCGACGCATTCCTGAT
*tdgcA*_212MR	AATCAGGAATGCGTCGCCGCCCC
*dgcF*_F	TGCA***<u>CCATGG</u>***GATTAAGGGACTTATTT
*dgcF*_R	CTGA***<u>CTCGAG</u>***AGATGCAGAGCTGACC
*tdgcF*_F	CTGA***<u>CCATGG</u>***AGTTATTTCAAAAGATGAAAC
*tdgcF*_DMF	CGAAAGGGTGGAGCGGCGTTTGCG
*tdgcF*_DMR	CGCAAACGCCGCTCCACCCTTTCG

[a] The restriction sites are underlined as ***bold Italic***.

Different strains containing single plasmids or double plasmids were transformed or co-transformed into corresponding *E. coli* strains (Table 1), and transformants were verified by sequencing. Plasmids were constructed as follows:

Construction of plasmids for β-galactosidase assay in *E. coli* Trans5α with various c-di-GMP riboswitch encoding regions in promotors

Gene *lacZ* was cut (fragments between *Hind* III and *Kpn* I) from our previously engineered plasmid pHT1K-*lacZ*(UTR)[3] and inserted into pRP1028 (a gift from Scott Stibitz, Center for Biologics Evaluation and Research, Food and Drug Administration, Bethesda, Maryland, USA) to generate pRP1028-*lacZ* (Supplementary Figure S6). Amplification of 5'-UTR encoding region fragments (including riboswitch encoding regions and their corresponding downstream sequences) *Bc5'* (primers: *Bc5*'_F, *5' UTR*_R), *Bc4-5'* (primers: *Bc4'*_F, *5' UTR*_R) and *Bc3-5'* (primers: *Bc3'*_F, *5' UTR*_R) were carried out using CT-43 genome DNA (GenBank accession: NC_017208.1)[4] as a template. Then they were linked (between *Nco* I and *BamH* I) into pRP1028-*lacZ* to generate plasmids pRP1028-*Bc5*'-*lacZ*, pRP1028-*Bc4-5*'-*lacZ* and pRP1028-*Bc3-5'*-*lacZ*, respectively (Supplementary Figure S6).

Construction of plasmids for PleD expression in *E. coli*

A previous reported DGC PleD[5] was used for the synthesis of intracellular c-di-GMP. Amplification of its encoding gene *pleD* (primer pair: *pleD*_F and *pleD*_R) was carried out using a gift PleD expression plasmid[6] as a template, and then fused (between *Xba* I and *Sal* I) into pET-28b(+) (Novagen, catalog

number: 69865-3) to form pET-28b(+)-*pleD* (Supplementary Figure S7). Subsequently, a fragment carrying P*tac* promoter was amplified (primer pair: P*tac*_F and P*tac*_R) from commercial plasmid pGEX-6P-1 (GE Healthcare Inc., code number: 28-9546-48) to replace the T7 promoter (between *Bgl* II and *Xba* I) of pET-28b(+)-*pleD*, thus the PleD constitutive expression plasmid pET-P*tac*-*pleD* (Supplementary Figure S7) was generated. Plasmid pET-28b(+) was used as a negative control.

Construction of riboswitch-based dual-fluorescence reporter plasmids used in *E. coli* BL21(DE3)

PleD inducible expression plasmid pET-28b(+)-*pleD* (Supplementary Figure S7) was constructed as above description and used for the regulation of intracellular c-di-GMP level, while pET-28b(+) was used as a negative control. Riboswitch-based dual-fluorescence reporter plasmids were constructed by fusing riboswitch encoding regions between *amcyan* (a cyan fluorescence protein gene from *Anemonia majano*) and *turborfp* (a turbo red fluorescence protein gene from *Entacmaea quadricolor*).

For construction of pRP0122-P*be*-*amcyan_Bc3-5_turborfp*, a backbone plasmid pRP0122 (Supplementary Figure S8) was firstly generated. In fact, pRP0122 was the truncated derivative of pRP1028, in which *turborfp* gene and P*be* were deleted. To obtain pRP0122, pRP1028 was cleaved with *Pvu* II and *EcoR* I to generate a linearized pRP1028, where the DNA region containing *turborfp* gene, spectinomycin resistance gene and P*be* was removed. Then, the spectinomycin resistance gene was amplified from pRP1028 using primers *speR*_F and *speR*_R (Supplementary Table S6) and ligated to the linearized pRP1028 at *Pvu* II and *EcoR* I sites, finally resulting in the formation of pRP0122. Subsequently, amplification of P*be* (primers: P*be*_F and P*be*_R), *amcyan* (primers: *amcyan*_F and *amcyan*_R), *Bc3-5* (primers: *Bc3*_F and *Bc5*_R) and *turborfp* (primers: *turborfp*_F and *turborfp*_R) were carried out using pRP1028, pSS4332, CT-43 genome DNA and pRP1028 plasmid as their corresponding templates, respectively. Plasmid pSS4332 was a gift from Scott Stibitz (Center for Biologics Evaluation and Research, Food and Drug Administration, Bethesda, Maryland, USA). Next, they were assembled with blunt ends through overlap extension PCR, then digested with *Kpn* I and ligated with a linearized pRP0122 which was cleaved with *Sma* I and *Kpn* I.

Amplification of *Bc3* (primers: *Bc3*_F and *Bc3*_R), *Bc3M* (primers: *Bc3M*_F and *Bc3*_R), *Bc4* (primers: *Bc4*_Fand *Bc4*_R), *Bc4M* (primers: *Bc4M*_F and *Bc4*_R), *Bc5* (primers: *Bc5*_F and *Bc5*_R), *Bc5M* (primers: *Bc5M*_F and *Bc5*_R), *Bc3-4* (primers: *Bc3*_F and *Bc4*_R) and *Bc4-5* (primers: *Bc4*_F and *Bc5*_R) were carried out using the CT-43 genome DNA as a template. Other c-di-GMP reporter plasmids pRP0122-P*be*-*amcyan_Bc3_turborfp*, pRP0122-P*be*-*amcyan_Bc3M_turborfp*, pRP0122-P*be*-*amcyan_Bc4_turborfp*, pRP0122-P*be*-*amcyan_Bc4M_turborfp*, pRP0122-P*be*-*amcyan_Bc5_turborfp*, pRP0122-P*be*-*amcyan_Bc5M_turborfp*, pRP0122-P*be*-*amcyan_Bc3-4_turborfp* and pRP0122-P*be*-*amcyan_Bc4-5_turborfp* were separately constructed by replacing *Bc3-5* of pRP0122-P*be*-*amcyan_Bc3-5_turborfp* with corresponding DNA fragments.

Construction of inducible expression plasmids for putative DGC overexpression and verification in *E. coli* BL21(DE3)

PleD inducible expression plasmid pET-28b(+)-*pleD* (Supplementary Figure S7) was constructed as above description and used as a positive control for putative DGCs verification. While pET-28b(+) was used as a negative control.

DgcA (XOO3988, GenPept accession: YP_202627.1) contains two transmembrane domains. So, its encoding gene *dgcA* and a truncated *dgcA* (*tdgcA*, encoding amino residues 75-296) were individually amplified from *Xanthomonas oryzae* pv. *oryzae* KACC 10331 genome DNA (GenBank accession: NC_006834.1)[7] using primer pairs *dgcA*_F/*dgcA*_R and *tdgcA*_F/*dgcA*_R and cloned into *BamH* I and *Hind* III of pET-28a(+) (Novagen, catalog number: 69864-3) and *Nco* I and *Xho* I of pET-28b(+) to obtain pET-28a(+)-*dgcA* and pET-28b(+)-*tdgcA*, respectively (Supplementary Figure S9). Subsequently, three tDgcA mutants encoding sequences: *tdgcA(D185A)*, *tdgcA(D211A)* and *tdgcA(E212A)* were separately obtained through overlap extension PCR using primer pairs *tdgcA*_185MF/*tdgcA*_185MR, *tdgcA*_211MF/*tdgcA*_211MR and *tdgcA*_212MF/*tdgcA*_212MR, and then cloned through the same procedure to generate pET-28b(+)-*tdgcA(D185A)*, pET-28b(+)-*tdgcA(D211A)* and pET-28b(+)-*tdgcA(E212A)*, respectively (Supplementary Figure S9).

DgcF (BMB171_C5008, GenPept accession: YP_003666238.1) contains five transmembrane regions. So, a truncated *dgcF* (*tdgcF*, encoding amino residues 187-352) was amplified from *B. thuringiensis* BMB171 genome DNA (GenBank accession: NC_014171.1)[8] with primers *tdgcF*_F and *dgcF*_R, and then inserted into pET-28b(+) between *Nco* I and *Xho* I to form pET-28b(+)-*tdgcF* (Supplementary Figure S10). A double-residues mutant *tdgcF(E273A, E274A)* was obtained through overlap extension PCR with a paired primers *tdgcF*_MF/*tdgcF*_MR, then cloned into pET-28b(+) between *Nco* I and *Xho* I to form pET-28b(+)-*tdgcF(E273A, E274A)* (Supplementary Figure S10).

The constructed plasmids were transformed to BL21(DE3) for Congo red assays and expressed proteins of putative DGCs and their variants were subsequently purified (Table 1). They are co-transformed to BL21(DE3) with pRP0122-P*be-amcyan_Bc3-5_turborfp* to construct the strains for putative DGCs verification (Table 1).

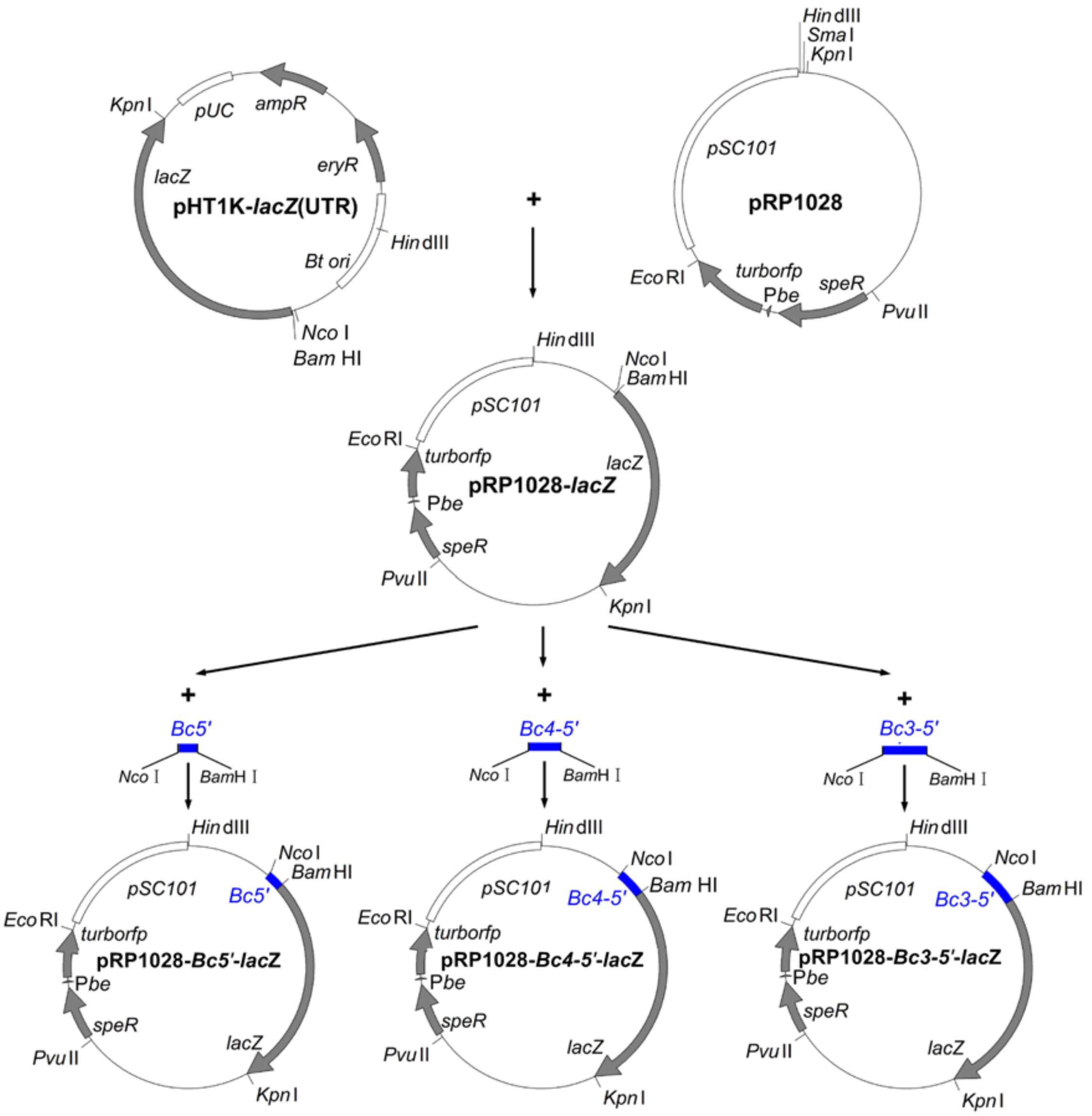

Figure S6. Construction of plasmids for β-galactosidase assay with various c-di-GMP riboswitch encoding regions inserted into promotors. The fragments *Bc5'*, *Bc4-5'* and *Bc3-5'* were truncated 5'-UTR encoding regions including Bc5, Bc4-5 and Bc3-5 RNA encoding regions and their corresponding downstream sequences; *lacZ*: *lacZ* translational fused with *spoVG* N-terminator; *speR*: spectinomycin resistance gene; P*be*: a region containing two family A promoters from *Bacillus* which is also active in *E. coli*; *pUC*: pUC replicon; *pSC101*: pSC101 replicon.

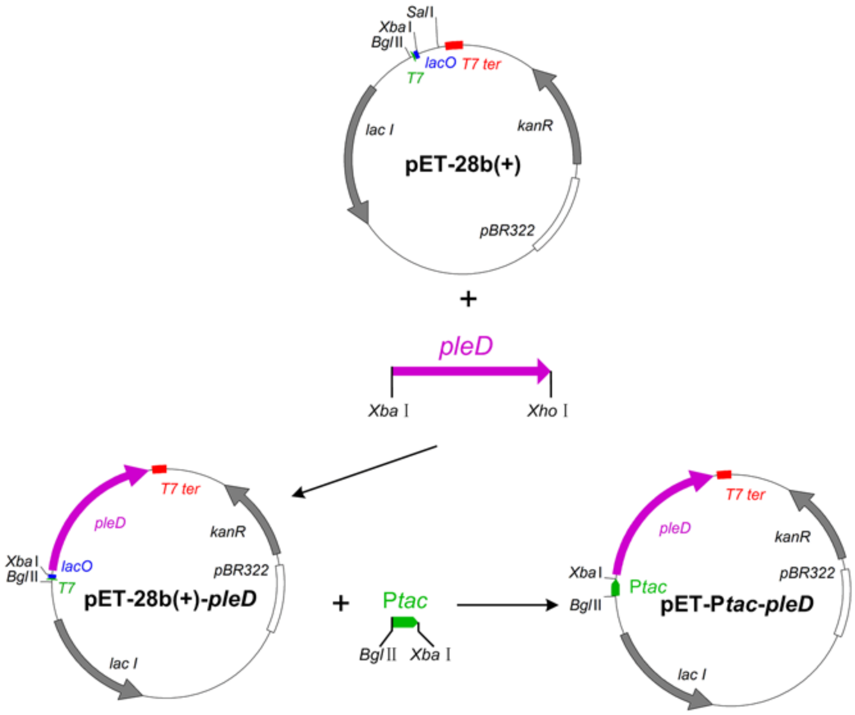

Figure S7. Construction of plasmids for PleD expression in *E. coli*. *pleD*: PleD encoding region; P*tac*: a DNA fragment containing P*tac* promoter; *T7*: T7 promoter; *lacO*: *lac* operator; *T7 ter*: T7 phage terminator; *kanR*: kanamycin resistance gene; *pBR322*: pBR322 replicon; *lacI*: lactose repressor gene. *Xho* I is an isocaudarner of *Sal* I.

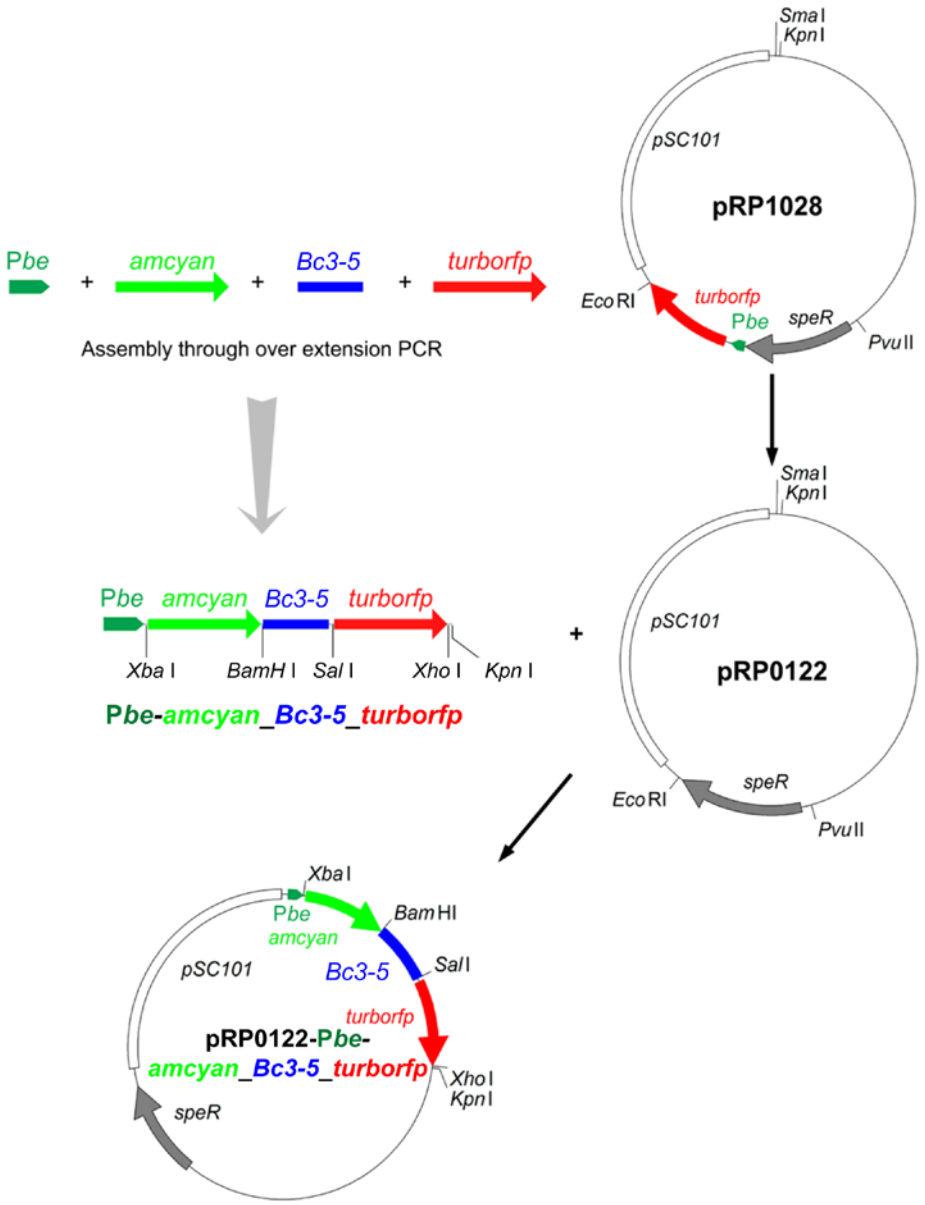

Figure S8. Schematic diagram of dual-fluorescent c-di-GMP reporter plasmid construction (taking pRP0122-P*be-cyan_Bc3-5_rfp* as an example). P*be*: a region containing two family A promoters from *Bacillus* is also active in *E. coli*; *amcyan*, *Bc3-5* and *turborfp* are encoding regions for AmCyan, triple-tandem c-di-GMP-I, and TurboRFP, respectively; *speR*: spectinomycin resistance gene; *pSC101*: pSC101 replicon. Other c-di-GMP reporter plasmids pRP0122-P*be-amcyan_Bc3_turborfp*, pRP0122-P*be-amcyan_Bc3M_turborfp*, pRP0122-P*be-amcyan_Bc4_turborfp*, pRP0122-P*be-amcyan_Bc4M_turborfp*, pRP0122-P*be-amcyan_Bc5_turborfp*, pRP0122-P*be-amcyan_Bc5M_turborfp*, pRP0122-P*be-amcyan_Bc3-4_turborfp* and pRP0122-P*be-amcyan_Bc4-5_turborfp* were separately constructed by replacing *Bc3-5* of pRP0122-P*be-amcyan_Bc3-5_turborfp* with corresponding DNA fragments.

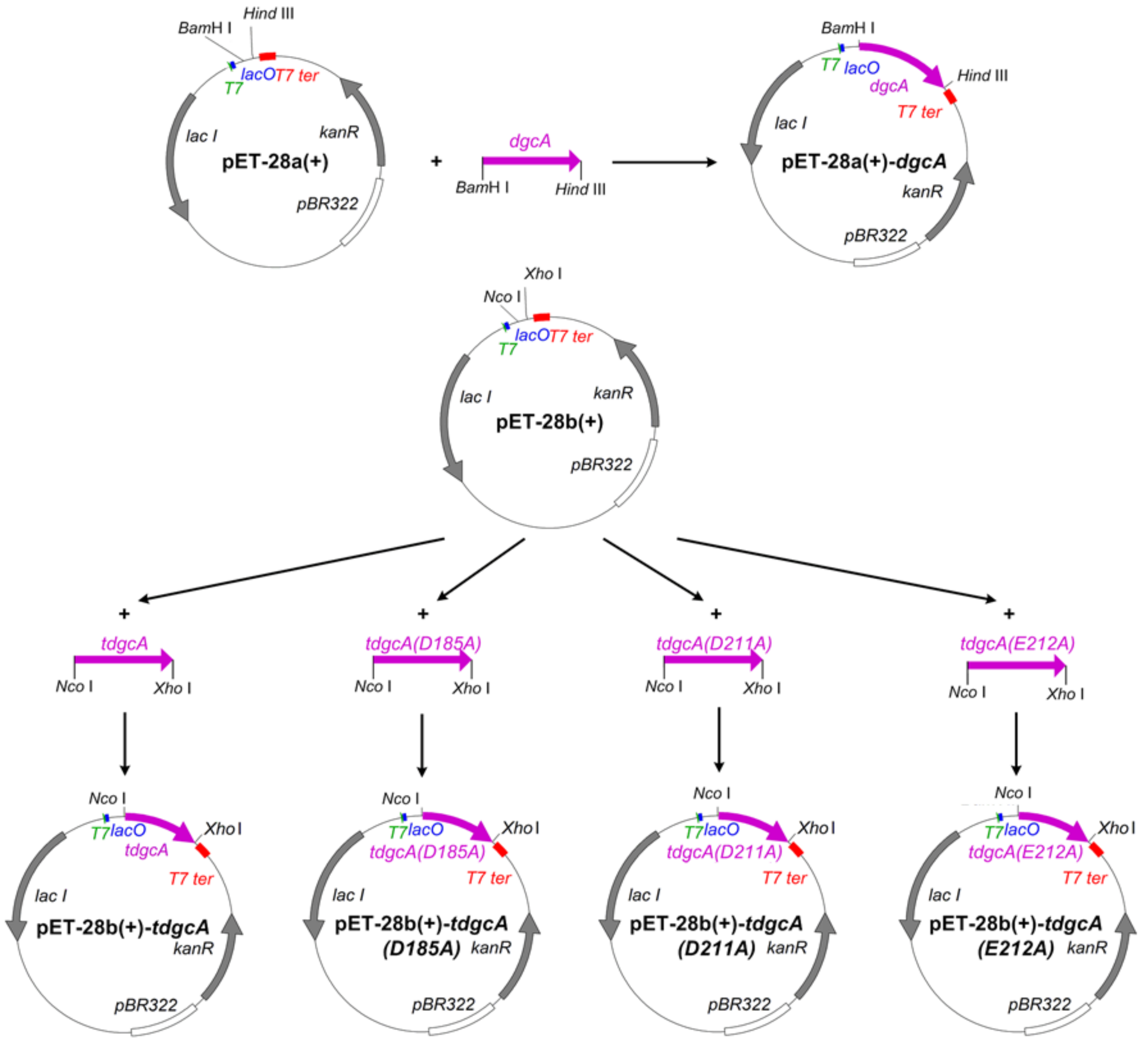

Figure S9. Construction of plasmids for heterogeneous expression of DgcA and its mutants in *E. coli* BL21(DE3). *dgcA*: DgcA encoding region; *tdgcA*: tDgcA encoding region; *tdgcA(D185A)*: tDgcA(D185A) encoding region; *tdgcA(D211A)*: tDgcA(D211A) encoding region; *tdgcA(E212A)*: tDgcA(E212A) encoding region; *T7*: T7 promoter; *lacO*: *lac* operator; *T7 ter*: T7 phage terminator; *kanR*: kanamycin resistance gene; *pBR322*: pBR322 replicon; *lacI*: lactose repressor gene.

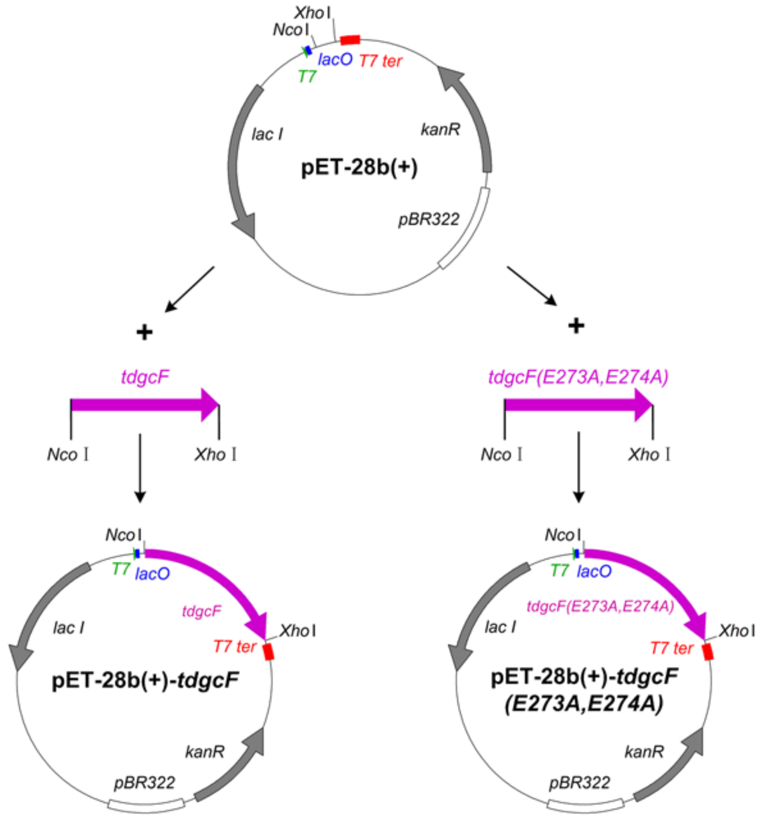

Figure S10. Construction of plasmids for heterogeneous expression of tDgcF and its double-site mutant in *E. coli* BL21(DE3). *tdgcF*: tDgcF encoding region; *tdgcF*(*E273A*, *E274A*): tDgcF(E273A, E274A) encoding region; *T7*: T7 promoter; *lacO*: *lac* operator; *T7 ter*: T7 phage terminator; *kanR*: kanamycin resistance gene; *pBR322*: pBR322 replicon; *lacI*: lactose repressor gene.

References

1. Brinster, S., Furlan, S. & Serror, P. C-terminal WxL domain mediates cell wall binding in *Enterococcus faecalis* and other gram-positive bacteria. *J. Bacteriol.* **189,** 1244-1253 (2007).
2. Navarre, W. W. & Schneewind, O. Proteolytic cleavage and cell wall anchoring at the LPXTG motif of surface proteins in gram-positive bacteria. *Mol. Microbiol.* **14,** 115-121 (1994).
3. Wang, J. *et al*. High-throughput identification of promoters and screening of highly active promoter-5'-UTR DNA region with different characteristics from *Bacillus thuringiensis*. *PLoS ONE* **8,** e62960 (2013).
4. He, J. *et al*. Complete Genome sequence of *Bacillus thuringiensis* subsp. *chinensis* strain CT-43. *J. Bacteriol.* **193,** 3407-3408 (2011).
5. Aldridge, P., Paul, R., Goymer, P., Rainey, P. & Jenal, U. Role of the GGDEF regulator PleD in polar development of *Caulobacter crescentus*. *Mol. Microbiol.* **47,** 1695-1708 (2003).
6. Chan, C. *et al*. Structural basis of activity and allosteric control of diguanylate cyclase. *Proc. Natl. Acad. Sci. USA* **101**, 17084-17089 (2004).
7. Lee, B. M. *et al*. The genome sequence of *Xanthomonas oryzae* pathovar *oryzae* KACC10331, the bacterial blight pathogen of rice. *Nucleic Acids Res.* **33,** 577-586 (2005).
8. He, J. *et al*. Complete genome sequence of *Bacillus thuringiensis* mutant strain BMB171. *J. Bacteriol.* **192,** 4074-4075 (2010).
